# Evaluation and comparison of large language models’ responses to questions related optic neuritis

**DOI:** 10.3389/fmed.2025.1516442

**Published:** 2025-06-25

**Authors:** Han-Jie He, Fang-Fang Zhao, Jia-Jian Liang, Yun Wang, Qian-Qian He, Hongjie Lin, Jingyun Cen, Feifei Chen, Tai-Ping Li, Zhanchi Hu, Jian-Feng Yang, Lan Chen, Carol Y. Cheung, Yih-Chung Tham, Ling-Ping Cen

**Affiliations:** ^1^Joint Shantou International Eye Center of Shantou University and The Chinese University of Hong Kong, Shantou, Guangdong, China; ^2^Shantou University Medical College, Shantou, Guangdong, China; ^3^Shaoguan University Medical College, Shaoguan, Guangdong, China; ^4^Dongguan Guangming Eye Hospital, Dongguan, Guangdong, China; ^5^Department of Ophthalmology and Visual Sciences, The Chinese University of Hong Kong, Hong Kong SAR, China; ^6^Yong Loo Lin School of Medicine, National University of Singapore, Singapore, Singapore; ^7^Centre of Innovation and Precision Eye Health, Department of Ophthalmology, Yong Loo Lin School of Medicine, National University of Singapore and National University Health System, Singapore, Singapore; ^8^Singapore Eye Research Institute, Singapore National Eye Centre, Singapore, Singapore; ^9^Guangdong Provincial Key Laboratory of Medical Immunology and Molecular Diagnostics, The First Dongguan Affiliated Hospital, School of Medical Technology, Guangdong Medical University, Dongguan, China

**Keywords:** eye diseases, optic nerve diseases, optic neuritis, artificial intelligence, natural language processing

## Abstract

**Objectives:**

Large language models (LLMs) show promise as clinical consultation tools and may assist optic neuritis patients, though research on their performance in this area is limited. Our study aims to assess and compare the performance of four commonly used LLM-Chatbots—Claude-2, ChatGPT-3.5, ChatGPT-4.0, and Google Bard—in addressing questions related to optic neuritis.

**Methods:**

We curated 24 optic neuritis-related questions and had three ophthalmologists rate the responses on two three-point scales for accuracy and comprehensiveness. We also assessed readability using four scales. The final results showed performance differences among the four LLM-Chatbots.

**Results:**

The average total accuracy scores (out of 9): ChatGPT-4.0 (7.62 ± 0.86), Google Bard (7.42 ± 1.20), ChatGPT-3.5 (7.21 ± 0.70), Claude-2 (6.44 ± 1.07). ChatGPT-4.0 (*p* = 0.0006) and Google Bard (*p* = 0.0015) were significantly more accurate than Claude-2. Also, 62.5% of ChatGPT-4.0’s responses were rated “Excellent,” followed by 58.3% for Google Bard, both higher than Claude-2’s 29.2% (all *p* ≤ 0.042) and ChatGPT-3.5’s 41.7%. Both Claude-2 and Google Bard had 8.3% “Deficient” responses. The comprehensiveness scores were similar among the four LLMs (*p* = 0.1531). Note that all responses require at least a university-level reading proficiency.

**Conclusion:**

Large language models-Chatbots hold immense potential as clinical consultation tools for optic neuritis, but they require further refinement and proper evaluation strategies before deployment to ensure reliable and accurate performance.

## 1 Introduction

Recent advancements in artificial intelligence (AI) have unlocked limitless possibilities for transforming medicine. Thanks to machine learning and deep learning technologies, AI has shown tremendous potential in healthcare (1). Currently, key applications of AI in medicine include enhancing interaction and communication, improving image recognition, supporting diagnostics and nursing, optimizing healthcare management and administrative processes, and assisting in surgeries and drug development.

Large language models (LLMs) are AI systems based on neural network architectures and use deep learning models, trained on extensive databases for natural language processing tasks. These models possess human-like language capabilities, offering substantial benefits to healthcare professionals and patients (2). Among them, ChatGPT, a generative AI developed by OpenAI (San Francisco, CA, United States), stands out for its widespread use as an AI chatbot by the general public. It leverages vast amounts of internet text data to produce coherent responses tailored to specific inputs (3). Unlike traditional search engines, ChatGPT and similar chatbots excel in simplicity, specificity, and interactivity, sparking increasing interest in their potential for medical consultations (4).

The application of large language models in ophthalmology is increasingly prevalent. Chatbots are utilized to assess proficiency in ophthalmology (4–8), educate clinical medical students, (9) assist in diagnostic processes for clinicians (10, 11), perform image diagnostics (12), manage clinical electronic records (13), educate patients (14) and aid in personalized patient management (14, 15). Notably, one study showed that ChatGPT achieved an accuracy rate above 90% on the Taiwanese medical licensing examination, yet it exhibited the highest error rate (28.95%) in ophthalmology-related questions (8). Moreover, while ChatGPT performed adequately in general ophthalmology queries, it showed weaknesses in neuro-ophthalmology and ocular pathology (5). The efficacy of LLM-Chatbots in addressing optic neuritis-specific questions remains unexplored. Given their potential role as assistants to doctors and patients, particularly in neuro-ophthalmology, there is a pressing need for further exploration of chatbots in this field.

Although the incidence of optic neuritis varies across regions and ethnic groups, it is reported that there are still about 4–8 per 100,000 person years globally (16). Due to the specialized expertise required to diagnose and treat optic neuritis, many patients struggle to access timely medical consultations with well-trained doctors. In such cases, more accessible alternatives like online consultations or chatbots become increasingly appealing (17). However, the effectiveness of chatbots in managing optic neuritis-related inquiries needs thorough evaluation.

In this study, we will compare the accuracy, comprehensiveness, and readability of four widely used and openly accessible LLM-Chatbots—ChatGPT-3.5, ChatGPT-4.0, Google Bard (now updated to Google Gemini), and Claude-2 (now updated to Claude-3) in clinical consultations for optic neuritis. Our findings will provide valuable insights into the effectiveness of LLM-Chatbots in clinical consultations for optic neuritis.

## 2 Materials and methods

In this study, we compared the accuracy, comprehensiveness, and readability of responses to optic neuritis-related questions generated by four LLM-Chatbots. The study was conducted from 23 January 2024 to 4 March 2024. Given that the study did not involve any patients or animals, approval from an ethics committee was not required.

### 2.1 Study design

Questions on optic neuritis were collaboratively developed by clinical ophthalmologists (LPC, HJH, FFZ), based on common issues faced by patients in clinical settings, frequently asked questions on online platforms, and authoritative information from esteemed health websites, such as the National Eye Institute and the American Academy of Ophthalmology (18, 19). Upon aggregating all relevant information, the three doctors, leveraging their clinical experience, consolidated 24 questions related to optic neuritis, categorized into four groups: general, diagnosis, treatment, and follow-up and prevention. This categorization aimed to assess the varied performance of different LLM-Chatbots across question types. For the study, conducted from 23 January to 30 January 2024, we utilized four LLMs: Claude-2 (Anthropic, San Francisco, California), ChatGPT (versions GPT-3.5 and GPT-4.0, OpenAI, San Francisco, California), and Google Bard (Google, Mountain View, California). While Claude-2, ChatGPT-3.5, and Google Bard are freely available, ChatGPT-4.0 requires a paid subscription. Nevertheless, given its enhanced performance in neuro-ophthalmology and its relative affordability and ease of use for patients, ChatGPT-4.0 was included in our study (5, 7).

Each of the 24 optic neuritis-related questions was directly entered into four LLM-Chatbots using separate, newly opened windows to prevent interference and preserve response integrity, with no specialized prompts employed in the process. A research member (HJH) uniformly collected all responses (Supplementary Tables 1–4), formatted them into plain text, and removed any identifiable features of each LLM-Chatbot without altering the main content. This ensured that evaluators could not determine which LLM-Chatbot produced the replies. To minimize potential biases across evaluations, three rounds of accuracy assessment were conducted, spaced 48 hours apart, with the sequence of responses rearranged before each round.

### 2.2 Evaluation of readability

Four validated readability scales (Supplementary Table 5) were used to assess responses from all LLM-Chatbots to optic neuritis-related questions (20–22), as well as authoritative online consultations accessible to patients, including content from the Mayo Clinic, Cleveland Clinic, and American Academy of Ophthalmology. These assessments utilized the Gunning Fog Index, Flesch-Kincaid Grade Level, Simple Measure of Gobbledygook (SMOG) score, and Coleman-Liau Index. Each of these scales measures word length, syntactic complexity, and sentence length, assigning a United States academic grade level necessary for comprehension. We calculated the readability scores for each response using a freely available online tool (23).

### 2.3 Evaluation of accuracy

The assessment team was made up of three neuro-ophthalmologists (FFZ, TPL, YW), each boasting at least 6 years of clinical experience. The assessment team was made up of three neuro-ophthalmologists (FFZ, TPL, YW), each boasting at least 6 years of clinical experience. Although the evaluators are non-native English speakers and may have certain shortcomings in understanding the linguistic nuances and cultural background of English content, the LLM-generated output on optic neuritis is predominantly medical (involving fewer complex cultural or idiomatic language issues), and importantly, all three hold medical master’s degrees and possess strong proficiency in ophthalmic medical English. They routinely use professional English in their clinical practice, which qualifies them to serve as evaluators. To ensure impartiality, these evaluators were not informed beforehand which LLM-Chatbot provided the responses. They independently assessed the accuracy of the replies using a three-point scale:

1. “Deficient”: Indicates responses that could significantly mislead and potentially harm patients due to inaccuracies.
2. “Marginal”: Signifies responses that contain possible factual errors, but with a lower risk of misleading or harming patients.
3. “Excellent”: Represents responses that are free from errors.

Ratings were determined based on a majority rule approach, where the responses from the LLM-Chatbots were assigned a rating after the majority rule was applied in each assessment round. If a discrepancy occurred among the three grading doctors’ opinions, the response was classified as “Pending.” After three evaluation rounds, each reply received three ratings. A final rating was established through the majority rule process (Supplementary Table 7). If this final rating remained “Pending,” a senior doctor (LPC) would then provide a conclusive rating.

### 2.4 Evaluation of comprehensiveness

Responses from chatbots deemed “Excellent” in accuracy will undergo further evaluation for comprehensiveness by the assessment panel. The evaluation utilizes a three-tiered scale:

1. Incomplete: Responses lack crucial key information necessary for completeness.
2. Comprehensive: Responses include all essential key information required.
3. Highly Comprehensive: Responses not only provide all key information but also include additional useful details that were not anticipated.

### 2.5 Statistical analysis

Statistical analyses for this study were conducted using GraphPad Prism (version 8.3.0). Descriptive statistics are presented as mean values and standard deviations (SD). For the parametric data, readability scores were analyzed using one-way ANOVA followed by Tukey’s multiple comparison post-hoc test across the four LLM-Chatbots and the overall web content. For non-parametric data, the Kruskal-Wallis Rank Sum test and Dunn’s multiple comparison post-hoc test were employed to evaluate the total accuracy and comprehensiveness scores across the four models. Additionally, a two-tailed Pearson’s χ^2^ was used to assess the distribution of accuracy ratings among the chatbots. The Bonferroni correction method was applied to adjust *p*-values for multiple comparisons, with a *p*-value below 0.05 considered statistically significant.

## 3 Results

### 3.1 Summary of response lengths

Table 1 presents the responses of all LLMs to optic neuritis questions. The average word count ± SD was: Claude-2 (220.29 ± 20.88), ChatGPT-3.5 (238.75 ± 71.36), Google Bard (299.75 ± 99.41), and ChatGPT-4.0 (269.25 ± 62.96). The average character count ± SD was: Claude-2 (1181.46 ± 140.96), ChatGPT-3.5 (1316.46 ± 410.83), Google Bard (1711.96 ± 576.22), and ChatGPT-4.0 (1461.63 ± 348.74). The average sentence count ± SD was: Claude-2 (15.42 ± 3.12), ChatGPT-3.5 (13.25 ± 4.08), Google Bard (15.46 ± 5.41), and ChatGPT-4.0 (13.83 ± 4.10).

**TABLE 1:** Overview of response length from large language models (LLM)-Chatbots to optic neuritis-related questions.

LLM	Response (words)			Response (characters)			Response (sentences)		
	Mean (SD)	Minimum	Maximum	Mean (SD)	Minimum	Maximum	Mean (SD)	Minimum	Maximum
Claude-2	220.29 (20.88)	171	258	1181.46 (140.94)	904	1453	15.42 (3.12)	11	22
ChatGPT-3.5	238.75 (71.36)	127	413	1316.46 (410.83)	729	2227	13.25 (4.08)	7	20
Google Bard	299.75 (99.41)	41	470	1711.96 (576.22)	214	2569	15.46 (5.41)	3	23
ChatGPT-4.0	269.25 (62.96)	144	390	1461.63 (348.74)	722	2096	13.83 (4.10)	7	26

### 3.2 Readability

**FIGURE 1:**
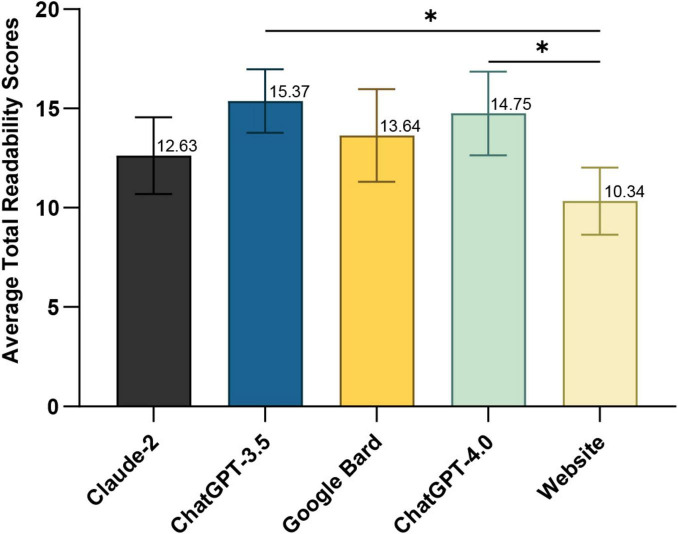
Average total readability scores of responses generated by large language models (LLM)-Chatbots and official website content. *P ≤ 0.05.

Figure 1 shows the average readability scores (Gunning Fog, Flesch-Kincaid, Coleman-Liau and SMOG; see Supplementary Table 6) for LLMs’ responses and professional web content on optic neuritis. The LLMs’ averages were: Claude-2, 12.47 ± 1.93; Google Bard, 13.64 ± 2.33; ChatGPT-4.0, 14.75 ± 2.11; and ChatGPT-3.5, 15.37 ± 1.60—all at the college level. The web content averaged 11.41 ± 1.73 (college level) (one-way ANOVA, *p* = 0.0188). ChatGPT-3.5 (Tukey’s *post hoc*, *p* = 0.0172) and ChatGPT-4.0 (Tukey’s *post hoc*, *p* = 0.0407) had significantly higher readability scores than the web content.

### 3.3 Accuracy

**FIGURE 2:**
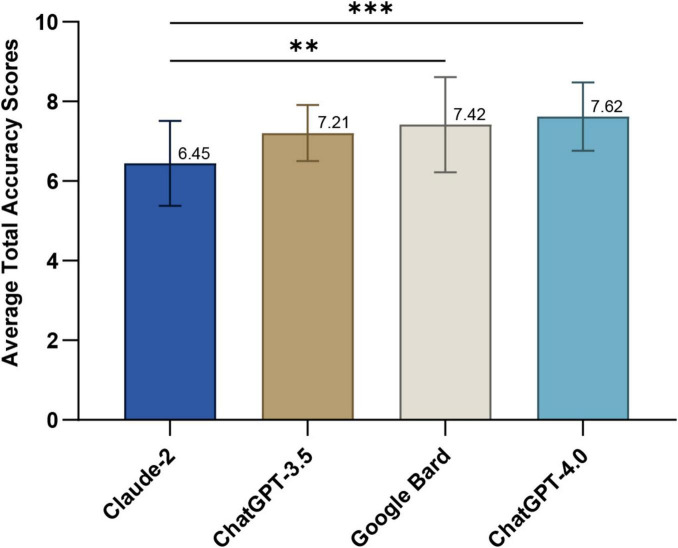
Average total accuracy scores of responses generated by large language models (LLM)-Chatbots. **P ≤ 0.01; ***P ≤ 0.001.

Figure 2 depicts the average overall accuracy scores for optic neuritis responses from each LLM, as rated by three neuro-ophthalmologists over three rounds. ChatGPT-4.0 scored highest (7.62 ± 0.86), significantly outperforming Claude-2 (6.44 ± 1.07, Dunn’s post-hoc test, p = 0.0006). Google Bard ranked second (7.42 ± 1.20; p = 0.0015 compared to Claude-2), followed by ChatGPT-3.5 (7.21 ± 0.70). Detailed scores for each question are in Supplementary Table 7.

**FIGURE 3:**
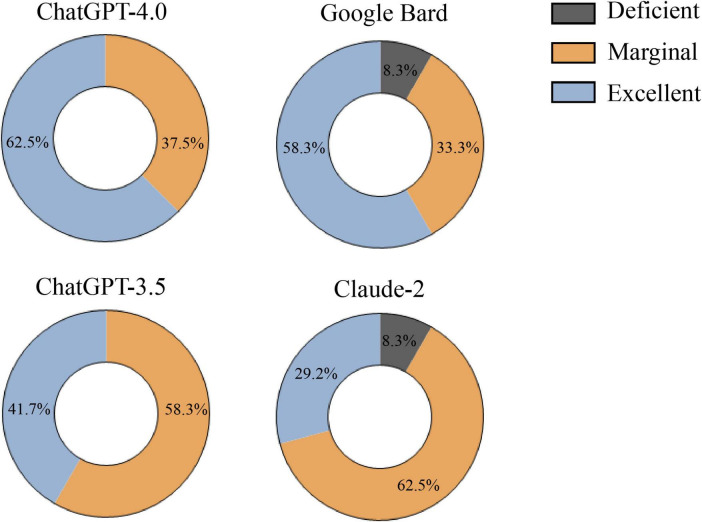
Final rating of responses generated by large language models (LLM)-Chatbots determined by the majority rule.

Figure 3 presents the final ratings for optic neuritis responses from each LLM after three rounds. ChatGPT-4.0 had 62.5% “Excellent” responses and Google Bard had 58.3%, both significantly higher than Claude-2’s 29.2% (Pearson’s chi-squared test, all *p* ≤ 0.042). ChatGPT-3.5 had 41.7% “Excellent.” “Deficient” ratings: 8.3% for Claude-2 and Google Bard, compared to 0% for both ChatGPT models. Detailed ratings for each LLM are in Supplementary Table 7.

Table 2 illustrates the rating distributions for LLMs’ responses to optic neuritis. ChatGPT-4.0 excelled in all categories (0% “Deficient”). ChatGPT-3.5 had no “Deficient” ratings but more “Marginal” ratings. Google Bard performed well in diagnosis and follow-up and prevention but had “Deficient” ratings in general and treatment. Claude-2 showed multiple Marginal ratings, two Deficient in treatment and no “Excellent” in follow-up and prevention.

**TABLE 2:** The percentage of ratings generated by each large language models (LLM)-Chatbots in different categories.

Category	Num-ber of quest-ions	Claude-2, *n* (%)			ChatGPT-3.5, *n* (%)			Google Bard, *n* (%)			ChatGPT-4.0, *n* (%)		
		Deficient	Marginal	Excellent	Deficient	Marginal	Excellent	Deficient	Marginal	Excellent	Deficie-t	Marginal	Excellent
General	6	0 (0)	4 (66.7)	2 (33.3)	0 (0)	3 (50)	3 (50)	1 (16.7)	2 (33.3)	3 (50)	0 (0)	2 (33.3)	4 (66.7)
Diagnose	5	0 (0)	3 (60)	2 (40)	0 (0)	3 (60)	2 (40)	0 (0)	1 (20)	4 (80)	0 (0)	2 (40)	3 (60)
Treatment	6	2 (33.3)	1 (16.7)	3 (50)	0 (0)	3 (50)	3 (50)	1 (16.7)	1 (16.7)	4 (66.7)	0 (0)	2 (33.3)	4 (66.7)
Follow-up and prevention	7	0 (0)	7 (100)	0 (0)	0 (0)	5 (71.4)	2 (28.6)	0 (0)	4 (57.1)	3 (42.9)	0 (0)	3 (42.9)	4 (57.1)

### 3.4 Comprehensiveness

Supplementary Table 8 shows “Excellent” response comprehensiveness scores. All chatbots performed similarly: Claude-2 (2.67 ± 0.34), ChatGPT-3.5 (2.43 ± 0.39), Google Bard (2.71 ± 0.34), and ChatGPT-4.0 (2.74 ± 0.19). No significant differences were found (Kruskal-Wallis test, *p* = 0.1531).

## 4 Discussion

Our study conducted a rigorous evaluation of four widely-used LLM-Chatbots—Claude-2, ChatGPT-3.5, Google Bard, and ChatGPT-4.0—on their handling of optic neuritis-related questions. We sourced common questions from multiple venues and had them input systematically into the chatbots by professional neuro-ophthalmologists. Responses were anonymized and randomized before being assessed across three rounds by experienced doctors, with senior doctors resolving any inconsistencies. Responses rated as “Excellent” were further examined for comprehensiveness. We also evaluated the readability of outputs from the LLM-Chatbots and established medical websites using an online tool. While previous research has explored LLM-Chatbots’ role in neuro-ophthalmology, such as producing patient handouts (24), comparing their responses with human experts on neuro-ophthalmology issues (25), and neuro-ophthalmic disease diagnosis (26), no prior studies have evaluated these chatbots on the three key aspects of accuracy, comprehensiveness, and readability for optic neuritis-specific questions—essentially what online patients need the most. Our findings could significantly enhance the use of LLM-Chatbots in neuro-ophthalmology, potentially establishing them as a new avenue for online consultations on optic neuritis, thereby underscoring our study’s substantial practical importance.

Regarding readability, both LLM-Chatbot responses and the content from accessible authoritative websites require a college-level reading proficiency, as indicated in Supplementary Table 6. This is considerably higher than the sixth-grade or lower level recommended by The American Medical Association (AMA) (27). This discrepancy echoes previous findings where online patient education materials (PEMs) on major ophthalmology websites significantly exceeded recommended reading levels (28). Poor readability of LLM-generated responses will diminish their utility in optic nerve clinical consultations, as patients who cannot comprehend the information—even if highly accurate—cannot benefit from it. For patients with lower health literacy, low-readability responses may lead to misunderstandings of medical information and even delay treatment. The relatively poor readability of LLM-Chatbots compared to standard PEMs may be attributed to the LLMs being trained on vast databases, including texts from specialized ophthalmology websites (1). Moreover, the highly specialized and somewhat niche nature of optic neuritis-related content means that LLMs trained with such information undoubtedly necessitate a higher reading level. Our research highlights the challenges LLMs face in balancing accuracy and readability. For instance, Claude-2 has lower accuracy but better readability, while ChatGPT-4.0 is the opposite. Contrary to other studies suggesting ChatGPT-4.0’s superior readability among LLMs, our findings suggest otherwise (20). Given that LLMs have the potential to simplify complex information, patients with lower educational levels could benefit by requesting simplified responses, thus maintaining content quality while making it more accessible (20, 22). This approach could be particularly useful for patients using LLM tools to address optic neuritis-related inquiries, guiding them in leveraging these technologies effectively.

In addressing questions related to optic neuritis, ChatGPT-4.0 demonstrates a significant advantage, achieving the highest average accuracy score and the most “Excellent” rated responses (Figures 2, 3). Google Bard closely follows, with performance nearly matching that of ChatGPT-4.0. ChatGPT-3.5 ranks in the middle, while Claude-2 shows the least favorable performance. Regarding comprehensiveness, the four LLM-Chatbots have successfully balanced accuracy and comprehensiveness, with their average scores all exceeding 2, thus achieving at least a “Comprehensive” rating (Supplementary Table 8). Our results corroborate earlier studies, indicating that ChatGPT-4.0 consistently outperforms other LLMs in the medical field, particularly in neuro-ophthalmology-related inquiries (5, 7, 29). The superior performance of ChatGPT-4.0 can be attributed to its enhanced model size and parameters, its expanding user base, and the incorporation of reinforcement learning from human feedback (RLHF), which helps in generating more relevant and contextually accurate responses (30, 31).

Our study reveals that while the majority of responses from the four models to optic neuritis-related questions were rated “Marginal” or better, Claude-2 and Google Bard each had responses categorized as “Deficient” in the general and treatment categories (Supplementary Table 7). This indicates that using LLMs to provide medical advice increases the risk of misleading information, especially for responses that are not rated as “Excellent.” Moreover, because most patients currently have access only to general-purpose LLMs, which have not received formal medical certification, their use may also raise legal and ethical concerns. For example, the clinical use of non-certified LLMs raises ethical concerns, because although LLMs are ethically prohibited from providing harmful information, erroneous medical advice may still indirectly harm patients, leading to delayed treatment or inappropriate self-medication. Given the swift advancement and intricate deployment of LLMs, the ethical challenges highlighted above are unlikely to be adequately safeguarded by current laws and regulations. Notably, Google Bard sometimes includes source links in its responses, but these links are often fabricated and lack authenticity (Supplementary Table 3). Previous research indicates that Google Bard has a tendency to generate fictitious or incorrect information (32), an issue that remains unresolved. Similarly, when ChatGPT-4.0 is prompted to provide sources, it might face the same problem (33). Therefore, caution is advised when considering the source information provided by LLMs.

Unlike traditional search engines, LLMs benefit from deep learning capabilities, continuously enhancing their knowledge from diverse online databases and user feedback. This highlights the significant potential of LLM-Chatbots in clinical settings. Numerous studies have explored LLM applications in various medical fields. For instance, Lim et al. (34) identified potential in handling consultations related to myopia, particularly with ChatGPT-4.0. Meng et al. (35) found that ChatGPT can provide appropriate responses to fracture prevention and medical queries. However, as Cappellani et al. (14) noted, ChatGPT can still generate incomplete, incorrect, or potentially harmful information about common ophthalmic diseases, reflecting the variable performance of LLMs across different medical fields. This variability is largely influenced by the specificity and development of those fields—the richer and more frequent the user interactions, the more effectively LLMs can learn and improve their performance.

Our study has several limitations. First, the small number of optic neuritis-related questions and the limited variety within categories might not fully represent the issues patients usually face, indicating a need for more diverse questions in future research. Additionally, to mitigate evaluator subjectivity, we used multiple evaluation rounds and majority rule decisions. While readability metrics help assess the educational level needed for understanding, they don’t encompass all comprehension factors. Lastly, the rapid evolution of LLMs, driven by new training data and user feedback, means our findings are time-sensitive, when new models emerge, longitudinal re-evaluation studies will be extremely valuable.

Our study confirms the potential of LLM-Chatbots to provide online clinical consultations for optic neuritis, offering accurate and comprehensive information across distances. However, their readability issues might affect user experience. More critically, any misinformation from LLM-Chatbots could lead to unforeseen harmful consequences. Patients using LLM-Chatbots need to proceed with caution and maintain open communication with their doctors, who in turn should guide their use of these tools effectively. Enhancing the readability, accuracy, and comprehensiveness of LLM-Chatbots is essential. A table summarizing the main findings can be found in Supplementary Table 9.

Future research should concentrate on refining assessment strategies for LLMs by developing more comprehensive scoring criteria. Additionally, ongoing training and targeted improvements are crucial to enhance the accuracy and readability of LLMs. Such efforts will ensure that their performance in addressing questions related to optic neuritis becomes increasingly robust and reliable.

## Data Availability

The original contributions presented in this study are included in this article/Supplementary material, further inquiries can be directed to the corresponding author.
