# Supplementary material for: Evaluation and comparison of large language models’ responses to questions related optic neuritis
Source: Front Med (Lausanne). 2025 Jun 25;12:1516442. doi: 10.3389/fmed.2025.1516442 (PMC12238082; doi:10.3389/fmed.2025.1516442)
Supplement: Supplementary file 1 [file Table_1.docx]

**Table S1**

**Claude-2**

1. **General**

| **Question** | **Response** |
| --- | --- |
| 1. What is optic neuritis, and how will it impact my vision? | Optic neuritis is inflammation of the optic nerve, which carries visual information from the eyes to the brain. It can cause vision loss, pain with eye movement, and impaired color vision.  Some key points about optic neuritis:  - It often affects only one eye, causing vision to become blurry, dim, or difficult to see from that eye. Vision may come and go.  - Pain is common, especially with eye movement. The eye itself is often sore to touch and movement may intensify the pain.  - Color vision is frequently impaired. Colors may appear "washed out" or difficult to distinguish.  - Contrast sensitivity and visual detail are also often reduced in the affected eye. Straight lines may appear warped or wavy.  - The exact cause is often unknown but it can be related to autoimmune disorders like multiple sclerosis or infections.  - Most people recover vision fully or almost fully within 10 weeks, especially if inflammation is treated early with IV steroids. However, some level of residual vision loss is possible.  - After an episode of optic neuritis, there is an increased risk for further vision loss, either in the same eye or the other eye, over time. Ongoing monitoring is beneficial.  In summary, optic neuritis causes inflammation of the optic nerve which can significantly, though often temporarily, impact vision. Prompt treatment is important to limit lasting damage and monitor for recurrence. Let me know if you have any other questions! |
| 1. Could you elaborate on the specific process leading to the formation of optic neuritis? | Here is a brief overview of the process leading to the formation of optic neuritis:  - Optic neuritis refers to inflammation of the optic nerve, which carries visual information from the eyes to the brain. It is often caused by an autoimmune reaction.  - In autoimmune optic neuritis, the body's own immune system mistakenly attacks the myelin sheath surrounding the optic nerve fibers. Myelin is an insulating layer made up of fats and proteins that allows nerve signals to transmit properly.  - The exact trigger for this autoimmune reaction is not always known, but it may be related to viral infections or other factors causing the immune system to turn against the myelin in the optic nerve. Certain genetic factors may also play a role.  - As inflammation damages the myelin, nerve signals from the eye slow down or cease to transmit along the optic nerve to the visual processing centers in the brain. Over time, axons (nerve fibers) may also become damaged.  - Signs and symptoms include vision loss or blurring in one or both eyes, pain with eye movement, dull ache behind the eye, loss of color vision, and difficulty seeing contrast.  - If untreated, episodes of inflammation can lead to permanent nerve damage and vision problems. That's why early diagnosis and treatment, like corticosteroids to reduce inflammation, are important in managing optic neuritis.  Let me know if you need any clarification or have additional questions! |
| 1. Is optic neuritis hereditary, and could I be at risk if it runs in my family? | Optic neuritis often occurs in association with certain autoimmune diseases like multiple sclerosis, which do tend to run in families to some degree. So in that sense, there can be a hereditary component. Specifically:  - If you have a close family member (parent, sibling, child) with multiple sclerosis, you have a slightly higher risk of developing MS and associated optic neuritis. The risk is estimated to be around 2-5%, compared to the general population risk of 0.1-0.3%.  - There are some genetic factors that can increase susceptibility to autoimmune conditions like MS. So if it runs strongly in your family, your personal genetic makeup may make you more prone to optic neuritis.  - However, optic neuritis can also occur in isolation or from other causes like viral infections. In those scenarios, family history would play less of a role.  The biggest risk factor is still having MS or other medical conditions that list optic neuritis as a symptom. But family history can contribute by increasing the chances you may develop those prime underlying diseases. If you have close relatives with optic neuritis or MS, make sure to let your doctor know so they can keep a closer eye out. But there are also many people with family histories who never go on to have issues. |
| 1. What are the characteristics of optic neuritis based on its types, and how might they impact me? | Optic neuritis has several types with differing characteristics and impacts:  - Acute demyelinating optic neuritis: Often associated with multiple sclerosis. Causes sudden vision loss, usually in one eye. Vision typically recovers within a few weeks but can have long-term mild vision impairment. High risk of developing MS.  - Chronic optic neuritis: Gradual vision loss over months/years, often in both eyes. Usually from diseases like sarcoidosis, syphilis, tuberculosis. Vision often continues declining without treatment of underlying condition.  - Neuromyelitis optica spectrum optic neuritis: Severe inflammation/damage of the optic nerve due to autoimmune disorder NMOSD. Causes rapid, often severe vision loss in one or both eyes over days/weeks. High risk of recurrence without treatment, can lead to blindness.  - Infectious optic neuritis: Result of infections like sinusitis, Lyme disease or cat scratch disease. Vision loss and pain similar to demyelinating ON, but vision can recover once infection treated.  - Compressive optic neuritis: Swelling of the optic nerve compressed in the orbit or optic canal. Causes progressive vision loss, looks similar to tumors/masses compressing the optic nerve.  The speed of onset, severity, whether one or both eyes affected, associated symptoms, risk of recurrence and the likelihood of vision recovery varies. Getting an exact diagnosis and treatment is important to prevent permanent vision damage. Let me know if you have any other specific questions! |
| 1. Are there any risk factors that could increase my chances of developing optic neuritis? | Here are some of the main risk factors that can increase the chances of developing optic neuritis:  - Multiple sclerosis - Optic neuritis often occurs as the first sign of MS. People with MS have a higher risk of optic neuritis.  - Viral or bacterial infections - Infections including measles, mumps, varicella-zoster (shingles), syphilis, tuberculosis, Lyme disease, and others may trigger optic neuritis.  - Autoimmune disorders - Diseases where the immune system attacks the body's own tissues can be associated with optic neuritis. These include lupus, sarcoidosis, and types of vasculitis.  - Vaccinations - While very rare, there have been some cases of optic neuritis developing after vaccinations for hepatitis B, measles/mumps/rubella, or influenza.  - Genetics - There may be inherited factors, as optic neuritis seems to run in some families. Certain genetic markers have also been linked with increased risk.  - Ethnicity - Optic neuritis seems to occur more commonly in Caucasian populations and less frequently in African American or Asian groups.  - Age and sex - Most cases occur in adults ages 18 to 45 years old. Women are affected about 3 times as often as men.  So being aware of these factors may help identify individuals at higher risk who should monitor visual symptoms closely. Rapid treatment is essential at the first sign of optic neuritis to limit damage and vision loss. Let me know if you need any clarification or have additional questions! |
| 1. What are the triggers for neuromyelitis optica, and how might they affect me? | Neuromyelitis optica (NMO) is an autoimmune disease that attacks the optic nerves and spinal cord. Some key things to know about potential triggers and impacts:  - Triggers are not fully understood, but may include infections, stress, pregnancy, and certain medications. Many people with NMO do not have clearly identified triggers prior to attacks.  - Attacks cause inflammation and damage in affected nerves. Common symptoms are eye pain/vision loss from optic neuritis and limb weakness/numbness from transverse myelitis.  - Recovery is often incomplete, so repeated attacks can lead to accumulating disability over time such as blindness or paralysis. Prompt treatment of attacks may limit damage.  - Around half of people with NMO will face some degree of permanent vision loss from optic nerve damage. Many require assistance with walking after spinal cord attacks.  - Steroid treatments during attacks and immunosuppressant medications in between can help reduce attack frequency and severity. Plasma exchange may also help stabilize acute attacks.  - If you are diagnosed with NMO, working closely with a neurologist familiar with the disease is recommended to develop a treatment plan suited to your situation. Let them know about any new symptoms between visits as well.  The uncertainty of NMO can be stressful, but understanding your options and triggers that affect you specifically can help regain a sense of control. Let me know if you have any other questions! |

1. **Diagnose**

| **Question** | **Response** |
| --- | --- |
| 1. How is optic neuritis diagnosed, and what signs should I look out for? | " Optic neuritis is typically diagnosed through a combination of patient history, eye exam, and possibly imaging tests or laboratory work. Some key signs and symptoms to look out for include:  - Vision loss - Usually starts in one eye. May be partial vision loss or complete blindness. Vision can fluctuate.  - Eye pain - Especially with eye movement. Pain is usually behind the eye rather than the eye surface.  - Decreased color vision - Colors may appear washed out or faded. Red colors are especially impacted.  - Uhthoff's phenomenon - Temporary worsening of vision and eye pain when the body temperature rises due to exercise, hot shower, fever, etc.  - Afferent pupillary defect (APD) - Unusual pupillary response when shining light in the affected eye, indicating nerve damage.  During an exam, the doctor will check visual acuity, color vision, pupil reactions, visual field, and examine the back of the eye. If optic neuritis is suspected, imaging tests like an orbital MRI may be done to evaluate the optic nerve. Tests of nerve function and inflammation could also be helpful. Prompt evaluation is important whenever optic neuritis is suspected. Early treatment can improve outcomes and minimize permanent damage. Let me know if you have any other questions!" |
| 1. What additional tests or examinations should I undergo to confirm and assess optic neuritis? | " Here are some additional tests that may be recommended to confirm and assess optic neuritis:  - Visual field testing - This checks for any blind spots or areas of vision loss. It helps locate the inflammation and damage in the optic nerve.  - Color vision testing - This can identify difficulties distinguishing colors, which may occur with optic neuritis.  - Visual evoked potentials (VEP) - Electrodes measure how your brain responds to visual stimuli. It can detect optic nerve damage and demyelination.  - Orbital MRI - An MRI with contrast dye provides detailed images of the optic nerve and surrounding structures to check for damage, lesions or abnormalities.  - Lumbar puncture (spinal tap) - This checks your cerebrospinal fluid for signs of inflammation and high white blood cell counts, which may indicate an autoimmune cause. It also helps rule out infections.  - Blood tests - Bloodwork looks for autoimmune disorders and vitamin deficiencies that may be associated with optic neuritis, as well as infections that can cause nerve inflammation.  - Optical coherence tomography (OCT) - This imaging test can reveal thinning of retinal nerve fibers caused by optic neuritis. It helps quantify nerve damage.  Let your ophthalmologist know if you experience any vision changes or worsening of symptoms so they can recommend appropriate diagnostic tests and treatments to prevent permanent vision loss. I hope this overview gives you a good starting point. Let me know if you need any clarification or have additional questions!" |
| 1. If I suspect I have optic neuritis, what steps should I take in seeking a diagnosis? | " Here are some recommended steps if you suspect you have optic neuritis:  1. See an ophthalmologist or neuro-ophthalmologist as soon as possible. Optic neuritis can lead to permanent vision loss, so getting a prompt diagnosis and treatment is important.  2. Be prepared to describe your symptoms in detail. This includes when the vision problems started, which eyes are affected, exactly what vision changes you are experiencing, and whether you have any pain with eye movement.  3. Get a comprehensive eye exam. This will likely include visual acuity testing, visual field testing, pupil examinations, eye movement examinations, and a fundus examination (to examine the back of the eye).  4. Be open to getting imaging tests. Optic neuritis is usually diagnosed partly based on results from MRI scans and other tests that can reveal inflammation or damage to the optic nerve. Common tests include MRI, optical coherence tomography (OCT), and visual evoked potentials.  5. Provide your medical history. Certain underlying conditions are associated with optic neuritis, so your doctor will want to know your medical background, including any previous neurological or autoimmune issues.  6. Follow treatment recommendations carefully. High doses of intravenous steroids are usually standard for treating acute optic neuritis to try to regain vision. Other immunosuppressant drugs may be considered as well.  Let your doctor guide the diagnostic process, but don't delay seeking attention if you notice vision changes that could suggest optic neuritis. Rapid treatment is key for the best outcome." |
| 1. What are the early symptoms that I might experience if I have optic neuritis? | " Here are some of the early symptoms that can occur with optic neuritis:  - Vision loss - This may start in one eye and be mild at first. You may notice blurred vision, darkened vision, or loss of color vision. Vision loss often gets worse over days or weeks.  - Eye pain - You may experience mild to severe pain with eye movement. The pain is typically behind the affected eye.  - Dimmed color vision - Colors may not appear as bright or vivid. Whites may appear washed out or gray.  - Floaters or flashes of light - You may see spots, floaters, flashes of light, or shadows in your field of vision.  - Difficulty seeing contrasts - Your ability to distinguish objects from their background may be reduced. Lines and edges may not stand out like normal.  - Swollen nerve - The optic nerve connecting your eye to your brain becomes inflamed. Your doctor can detect this during an eye exam.  If you develop any concerning vision symptoms that persist or worsen, it's important to see an ophthalmologist or neurologist to get evaluated for possible optic neuritis or other conditions affecting the optic nerve. Early treatment can help prevent permanent damage and vision loss. Let me know if you have any other questions!" |
| 1. Why is a comprehensive examination necessary for diagnosing optic neuritis? | " An optic neuritis comprehensive exam is important for several reasons:  1. To confirm the diagnosis and rule out other possible causes of vision loss. An exam will assess visual acuity, visual fields, color vision, pupil responses, eye movements, and examine the retina and optic nerve for signs of inflammation or damage.  2. To determine if it is related to multiple sclerosis. Around 20-50% of optic neuritis cases represent a first attack of MS. Exam findings, patient history, and MRI scans can help predict MS risk.  3. To assess severity and monitor progression. Serial visual field testing, visual acuity checks, and optical coherence tomography can quantify nerve damage and monitor changes over time.  4. To establish a treatment plan. Steroid treatment helps shorten the duration and reduce severity of an episode of optic neuritis. Knowing exam findings helps determine if/when treatment is warranted.  5. To provide a baseline for future comparisons. Documenting clinical exam findings creates a baseline to which future exams can be compared to assess recovery or worsening.  In summary, a thorough diagnostic workup, including a comprehensive eye exam, imaging, and lab tests, is vital for understanding the underlying cause, severity, and prognosis when optic neuritis is suspected. It guides immediate treatment decisions and long-term monitoring." |

1. **Treatment**

| **Question** | **Response** |
| --- | --- |
| 1. What treatment options are available for optic neuritis, and how effective are they? | " Here is a summary of the main treatment options for optic neuritis and their effectiveness:  - Corticosteroids (e.g. intravenous methylprednisolone) - These are often the first line of treatment to reduce inflammation and speed up recovery of vision. Multiple studies have shown corticosteroids lead to faster vision recovery, though may not affect long-term outcomes.  - Plasma exchange - This involves removing antibodies/toxins from the blood. Some research indicates plasma exchange followed by corticosteroids can quicken vision recovery compared to corticosteroids alone. But results are mixed.  - Immunosuppressants - Drugs like azathioprine, rituximab, and natalizumab may be used, especially for recurrent or severe optic neuritis. They suppress the immune system to limit nerve damage from inflammation. Evidence on their efficacy is limited but promising for improving vision outcomes.  - Rehabilitation - Vision rehabilitation training, occupational therapy, and low vision aids may help some patients adapt and maximize remaining vision during recovery. Controlled trial data is limited but shows possible quality of life benefits.  In general, the earlier treatment begins after onset of optic neuritis symptoms, the better the visual prognosis. Even with treatment, full vision recovery is not guaranteed and optic neuritis can recur in some patients. More research is still underway on optimizing management strategies." |
| 1. Can optic neuritis be naturally eliminated, and how can I increase the chances of that happening? | " Unfortunately, optic neuritis usually does not resolve on its own. Here are some tips that may help improve the chances of recovery:  - Get treated early. The sooner treatment is started with IV steroids, the better the chances of regaining vision. Don't delay seeking medical care.  - Take steroid treatment as prescribed. Oral or intravenous steroids reduce inflammation and may help preserve nerve function. Completing the full course as directed is important.  - Rest your eyes. Give them a break from visually intensive tasks while recovering. Avoid driving until cleared by your doctor.  - Manage underlying causes. If optic neuritis is related to an autoimmune disorder like multiple sclerosis, properly treating that condition may reduce flare-ups.  - Remain optimistic. Many people regain most or all of their vision within a few weeks or months after an optic neuritis episode. Stay positive and follow your treatment plan.  - Get enough key nutrients. Eat a healthy diet and supplement if directed by your doctor to support nerve healing. Key nutrients include vitamins B1, B12, D, E, omega-3s, and antioxidants.  The nerves have a capacity to heal over time, especially when supported through proper medical treatment and healthy lifestyle factors. Work closely with your doctor for the best recovery. While spontaneous full recovery is unlikely, the prognosis can be good with early treatment." |
| 1. How can I relieve pain and other symptoms caused by optic neuritis? | " Here are some tips that may help relieve optic neuritis symptoms:  - Rest your eyes and avoid visually demanding tasks. Close your eyes periodically and use artificial tears if your eyes feel dry or strained.  - Take over-the-counter pain medications like acetaminophen or ibuprofen to relieve eye pain and headache.  - Use a warm or cold compress on your eyes to reduce pain and swelling.  - Take prescribed steroid medication (oral or intravenous) to reduce inflammation and speed up recovery.  - Consider asking your doctor about nerve pain medications if you have severe eye pain. Medications like gabapentin or amitriptyline may provide relief.  - Use sunglasses, even indoors, if bright light exacerbates your symptoms. Blue light filtering lenses can also help.  - Drink plenty of fluids and get lots of rest. Fatigue can worsen optic neuritis symptoms.  - Consider vision rehabilitation with a specialist once acute symptoms resolve. This can aid recovery.  Let your doctor know right away if symptoms worsen or you experience sudden vision loss. Prompt treatment is important for preserving vision with optic neuritis." |
| 1. Is it possible for my vision to fully recover to normal after optic neuritis treatment? | " Yes, it is often possible for vision to fully recover after optic neuritis treatment. Here are some key points about recovery:  - With treatment, vision usually begins improving within a few weeks, though full recovery can take up to a year or sometimes longer.  - Up to 70-80% of people regain 20/20 or near 20/20 vision after one optic neuritis episode when treated properly. The quicker treatment begins, the better the chances.  - Even if some visual loss remains, further recovery is still possible for months or years after the initial inflammation has resolved.  - Factors that influence individual prognosis include the severity of inflammation, whether it was a first or recurring episode, the presence of multiple sclerosis or other conditions, age, and how quickly treatment was initiated.  - Treatments aim to reduce inflammation and may include IV steroids, plasma exchange, or immunosuppressants. Most patients also transition to maintenance meds like interferons.  - Remaining vision deficits may continue improving gradually over time. Low vision rehab and visual field recovery therapy can also help continue progress.  So in summary - yes, full vision recovery is often attainable, especially with prompt treatment and follow-up rehabilitation care. Working closely with your neuro-ophthalmologist is key to monitoring and supporting ongoing healing. Let me know if you have any other questions!" |
| 1. What potential risks are associated with corticosteroid therapy for optic neuritis? | " Some potential risks associated with corticosteroid therapy for optic neuritis include:  - Exacerbation of infection - Corticosteroids suppress the immune system, which could worsen an existing infection or increase susceptibility to new infections. Care should be taken in patients with infections before starting corticosteroids.  - Gastrointestinal issues - Corticosteroids can cause nausea, vomiting, and ulcers in the stomach and intestines. Proton pump inhibitors or H2 blockers may need to be co-prescribed to reduce this risk.  - Mood disturbances - Steroid therapy has been associated with mood swings such as mania, depression, and psychosis in some patients. Patients should be monitored for any mental status changes.  - Fluid retention - Steroids can cause the body to retain salt and water, leading to weight gain, ankle swelling, and high blood pressure. A low salt diet and diuretics may be utilized to minimize fluid retention.  - Osteoporosis - Increased risk of osteoporosis and fractures with long-term corticosteroid therapy. Supplementation with calcium and vitamin D may help counteract bone loss.  - Impaired wound healing - Steroids slow the rate of healing after injuries and surgery. The timing of any elective procedures may need to be deferred until after a course of steroids.  - Eye issues - Specifically for optic neuritis, steroids carry risks of increased intraocular pressure and cataracts. Regular eye exams to monitor for these complications are recommended.  The decision to use corticosteroid therapy involves weighing these risks against the potential benefits in speeding recovery in optic neuritis. Careful patient selection and monitoring can help mitigate some of these risks." |
| 1. When is the optimal time to begin treating optic neuritis, and what happens if treatment is delayed? | " Optic neuritis refers to inflammation of the optic nerve that often causes vision loss. Here are some key points about when to start treatment and what can happen with delayed treatment:  - It is generally recommended to start high-dose intravenous corticosteroid treatment as soon as possible after diagnosing optic neuritis. Many ophthalmologists and neurologists recommend starting within 1-2 weeks.  - Beginning treatment quickly aims to hasten recovery, reduce the severity of vision loss, and lower the risk of progression to multiple sclerosis (MS) in those with acute demyelinating optic neuritis.  - Delaying corticosteroid treatment beyond 3-4 weeks is associated with poorer visual outcomes. Individuals who do not receive treatment have 2-3 times higher risk of severe permanent vision impairment in the affected eye.  - Untreated, 70-90% of optic neuritis patients experience some level of spontaneous visual recovery. However, delaying reduces the level and pace of recovery. Around 30% have 20/40 vision or worse after 1 year if untreated.  - Beyond vision outcomes, earlier treatment is also important for reducing likelihood of conversion to definite MS diagnosis down the road and for preserving quality of life related to vision in the near-term.  In summary, immediately to within a few weeks is considered the optimal and recommended timeframe to start corticosteroid therapy after diagnosing optic neuritis. Delaying treatment risks poorer visual outcomes as well as higher subsequent MS risk." |

1. **Follow-up and Prevention**

| **Question** | **Response** |
| --- | --- |
| 1. Does optic neuritis commonly recur, and if so, which type has a higher recurrence rate? | " Optic neuritis often occurs as a single, isolated episode, but recurrence is not uncommon. Some key points about recurrent optic neuritis:  - Recurrent optic neuritis is more commonly seen with certain conditions such as multiple sclerosis (MS), neuromyelitis optica spectrum disorder (NMOSD), or chronic relapsing inflammatory optic neuropathy (CRION).  - Isolated, idiopathic optic neuritis has a relatively low recurrence rate of around 10-25% over 10-15 years. The risk may be slightly higher in children.  - Optic neuritis associated with NMOSD tends to have the highest recurrence rates, reported between 50-75% at 5 years. This is because NMOSD is a relapsing autoimmune condition targeting the nerves including the optic nerve.  - In MS-associated optic neuritis, recurrence rates vary widely from 10-57% in different studies over 5-15 years. Higher recurrence rates may be seen with more active/severe MS disease.  So in summary, optic neuritis recurrence is not uncommon, especially with underlying autoimmune conditions. NMOSD-related optic neuritis seems to recur more frequently than MS or isolated types. Careful diagnosis of underlying causes guides expected prognosis. Monitoring and preventative treatment after an initial episode may help reduce future recurrences." |
| 1. What preventive measures can I take to avoid developing optic neuritis? | " Here are some tips to help prevent or reduce the risk of developing optic neuritis:  - Get treated promptly for viral and bacterial infections. Infections like sinusitis, COVID-19, or the flu can sometimes trigger optic neuritis. Treating them quickly may prevent it from developing.  - Carefully manage autoimmune conditions. Diseases like multiple sclerosis and neuromyelitis optica can cause optic neuritis episodes. Following your treatment plan closely to keep the condition well-controlled may lower your risk.  - Prevent vitamin B12 deficiency. Low vitamin B12 is a known risk factor. Eat foods high in B12 like meat, eggs, and dairy or take supplements if deficient. Getting bloodwork periodically can help detect a deficiency early.  - Wear protective eyewear. Protecting your eyes from blunt injuries can prevent traumatic optic neuritis. Wear safety glasses or goggles if participating in activities with flying debris.  - Quit smoking. Some studies link tobacco use to higher odds of developing optic neuritis. Quitting can not only improve eye health but overall health.  - Follow up on unusual vision changes. See an ophthalmologist or neuro-ophthalmologist right away if you notice new visual disturbances, eye pain, vision loss or floaters to determine if optic neuritis is developing. Early treatment provides the best outcomes.  Implementing some of these prevention tips may reduce the chances of optic neuritis occurring or minimize severity. However, even with precautions some cases may still develop due to individual risk factors." |
| 1. How should I proceed with follow-up care after experiencing optic neuritis? | " Here are some tips for follow-up care after experiencing optic neuritis:  - See an ophthalmologist and neurologist for ongoing monitoring. It's important to have regular check-ups to assess vision changes and see if the inflammation is recurring.  - You may need to do visual field testing every 6 months or so to check your peripheral vision. This helps detect any new blind spots.  - Get a baseline MRI scan soon after the initial attack. Further testing can reveal if you have MS or other conditions that caused the optic neuritis.  - Take recommended supplements and medications. Your doctors may suggest high-dose steroids to reduce inflammation during acute attacks. Low-dose antidepressants may help with nerve pain.  - Contact your doctor right away if vision worsens or you experience new neurological symptoms. This could indicate another attack or progression of an underlying condition.  - Ask your doctors about steps to recover visual acuity or adaptation therapies if you are left with permanent vision loss. Low vision rehabilitation can help strengthen usable vision.  - Maintain peripheral vision by scanning your full visual field. Try not to over-focus centrally. Turn your head to see side-to-side.  - Get plenty of rest and avoidtriggers like stress that can exacerbate autoimmune dysfunction. Stay active but listen to warning signs from your body.  The key is managing long-term nerve health and function. Report any new vision or neurological changes promptly. Consistent monitoring and restorative therapies can aid recovery. Let me know if you need any other optic neuritis management tips!" |
| 1. What steps can be taken to prevent the recurrence of neuromyelitis optica? | " Here are some steps that can help prevent relapses or recurrence of neuromyelitis optica (NMO):  1. Adhere strictly to immunosuppressant medications as prescribed. Medications like rituximab, azathioprine, mycophenolate, and steroids help prevent relapses by suppressing the immune system attacks on the optic nerves and spinal cord.  2. Pay close attention to early signs and symptoms of a relapse. Symptoms like eye pain/vision loss, weakness/numbness in limbs, and bladder/bowel problems could indicate an impending attack. Prompt treatment can limit damage from a relapse.  3. Avoid potential triggers that can activate the immune system. Things like infections, physical trauma, pregnancy, and certain medications are suspected of increasing relapse risks for some NMO patients. Stay vigilant about avoiding triggers relevant to your situation.  4. Adopt a healthy lifestyle and stress management routine. Maintain a nutritious diet, exercise program, adequate sleep and rest, and relaxation techniques to support the immune system functioning optimally.  5. Get vaccinated for preventable infections. Making sure your routine vaccinations are up-to-date can help avoid infections that could provoke an NMO attack. The flu shot and COVID vaccines are especially important.  6. Have regular follow-up and monitoring with your NMO specialist. Through ongoing lab work and check-ups, your doctor can closely supervise your condition for signs of returning disease activity. This allows adjusting treatment as needed to maintain remission.  The key is working closely with your healthcare providers to find the right long-term treatment plan to keep NMO flares under control. Stay vigilant and don't hesitate to report any new symptoms or concerns." |
| 1. In the event of a recurrence of neuromyelitis optica, what actions should I take? | " Here are some recommended steps to take in the event of a neuromyelitis optica (NMO) recurrence:  - Seek medical attention immediately. Recurrences of NMO symptoms can cause serious neurological damage and blindness if not treated quickly. Alert your doctor about any new or recurring symptoms.  - Get retested for NMO antibodies. A recurrence may indicate your treatment needs to be adjusted to better suppress the antibodies causing the nerve inflammation.  - Consider starting or adjusting immunosuppressant medications as prescribed by your doctor to help prevent further relapses. Medications like rituximab, azathioprine, mycophenolate, and steroids may be used.  - Adhere closely to your treatment plan as recommended by your neurologist. Make sure you understand what medications you need to take, their dosages, and any lifestyle changes suggested.  - Rest and allow yourself time to recover. NMO episodes can be physically and mentally draining. Give your body what it needs to help nerves heal.  - Seek supportive care like physical, occupational or speech therapy if you have new functional disabilities. Therapists can help you adapt and recover.  The key is getting professional care quickly at the onset of any new symptoms to limit Nerve damage from inflammation and relapse. Stay in close contact with your specialist. Let me know if you need any other specific NMO advice or information." |
| 1. What potential consequences might arise from multiple recurrences of neuromyelitis optica? | " Here are some potential consequences that may arise from multiple recurrences of neuromyelitis optica (NMO):  - Progressive vision loss or blindness. NMO frequently causes inflammation/damage to the optic nerves, and repeated attacks can lead to cumulative damage and permanent vision impairment over time.  - Progressive motor weakness or paralysis of limbs. Recurrent spinal cord inflammation from NMO episodes can lead to accumulating damage, loss of motor function, and paralysis in the legs or arms.  - Neuropathic pain. The nerve inflammation and damage can also result in chronic neurogenic pain signals from the affected areas.  - Bladder/bowel dysfunction. Inflammation in the spinal cord can impact nerve signaling to the bladder and bowel, resulting in loss of normal function like incontinence or impairment of normal elimination.  - Respiratory failure. If high spinal nerve inflammation reaches the cervical (neck) spinal regions, it can weaken the diaphragm and intercostal muscles, which are critical for breathing.  - Ongoing autoimmune disease progression. People with recurring bouts of NMO often have worsening autoimmune activity over time, including developing more autoreactive antibodies/immune cells or spreading inflammation to additional regions. This can lead to accumulating disability.  - Emotional/psychological effects like depression. The recurrent bouts of severe symptoms, accumulating disabilities, visual loss, and pain can lead to significant emotional and psychological tolls over the long-term course of the disease. Support is often needed.  The overall result can be severe impairment and disability if the NMO recurrences continue unchecked and untreated. Early, aggressive treatment to slow or stop attacks is critical." |
| 1. Can individuals with neuromyelitis optica safely receive vaccinations? | " Yes, individuals with neuromyelitis optica (NMO) can generally receive vaccinations safely, but there are some key points to consider:  - Inactivated vaccines, like the flu shot, are considered safe for people with NMO. These vaccines don't contain any live virus and therefore don't trigger disease activity.  - Live attenuated vaccines, which do contain weakened forms of live viruses, have the potential risk of triggering an autoimmune flare or relapse in people with NMO and some other autoimmune diseases. Examples include measles, mumps and rubella (MMR), varicella (chickenpox) and intranasal influenza vaccines. These are usually avoided.  - It's recommended that vaccinations, if possible, should be administered during times of remission rather than active disease for individuals with NMO.  - People on immunosuppressive medications for NMO should avoid most live vaccines, as their immune response may be diminished. Inactivated vaccines can still typically be given, but their efficacy could potentially be slightly lower.  So the current clinical guidelines support the use of inactivated, non-live vaccines for those with neuromyelitis optica. Having discussions with one's neurologist is important when making vaccine decisions. Ensuring one's immune system isn't already suppressed from medications or active disease is also key for an appropriate vaccine immune response. But vaccinations are an important preventative tool that are encouraged when they can be safely administered." |
