# Supplementary material for: Evaluation and comparison of large language models’ responses to questions related optic neuritis
Source: Front Med (Lausanne). 2025 Jun 25;12:1516442. doi: 10.3389/fmed.2025.1516442 (PMC12238082; doi:10.3389/fmed.2025.1516442)
Supplement: Supplementary file 2 [file Table_2.docx]

**Table S2**

**ChatGPT-3.5**

1. **General**

| **Question** | **Response** |
| --- | --- |
| 1. What is optic neuritis, and how will it impact my vision? | Optic neuritis is the inflammation of the optic nerve, which is the bundle of nerve fibers that transmit visual information from the eye to the brain. This inflammation can cause a variety of symptoms and may affect vision in one or both eyes.  Common symptoms of optic neuritis include:  1. Blurred vision: Vision may become blurred, and objects may appear hazy.  2. Loss of color vision: Some people with optic neuritis experience a decrease in their ability to perceive colors.  3. Pain: Pain, often described as a sharp or stabbing pain, may occur with eye movement. However, not everyone with optic neuritis experiences pain.  4. Visual field loss: There may be a partial or complete loss of vision in one eye or both eyes, usually affecting the peripheral vision.  The severity and impact on vision can vary from person to person. Optic neuritis is often associated with demyelinating diseases such as multiple sclerosis (MS), where the protective covering of nerve fibers (myelin) is damaged. However, optic neuritis can also occur independently.  The exact cause of optic neuritis is not always clear, but it is thought to involve an immune system attack on the optic nerve. It is more common in young adults, particularly women.  Treatment for optic neuritis often involves addressing the underlying cause, if known. In some cases, corticosteroids may be prescribed to reduce inflammation and speed up recovery. However, the outlook for optic neuritis is generally favorable, and many people experience significant improvement in their vision over time. It's essential to consult with a healthcare professional for a proper diagnosis and appropriate management based on your specific situation. |
| 1. Could you elaborate on the specific process leading to the formation of optic neuritis? | Optic neuritis is a condition characterized by inflammation of the optic nerve, which is the nerve that transmits visual information from the eye to the brain. The exact cause of optic neuritis is not always clear, but it is often associated with autoimmune or demyelinating processes. Here is an overview of the process leading to the formation of optic neuritis:  1. Autoimmune Response: In many cases, optic neuritis is believed to result from an autoimmune response, where the immune system mistakenly attacks the myelin sheath that covers the optic nerve. Myelin is a fatty substance that insulates and protects nerve fibers, facilitating the transmission of electrical signals.  2. Demyelination: The immune system's attack on the optic nerve leads to inflammation and demyelination. Demyelination refers to the loss or damage of the myelin sheath. As a result, the transmission of signals along the optic nerve becomes impaired.  3. Swelling and Compression: Inflammation in the optic nerve can cause swelling, leading to compression of the nerve fibers. This compression further hampers the transmission of visual signals, causing symptoms such as vision loss, blurred vision, or pain with eye movement.  4. Nerve Damage: Over time, if the inflammation and demyelination persist, it can lead to permanent damage to the optic nerve fibers. This may result in long-term visual impairment or even blindness in severe cases.  5. Underlying Conditions: Optic neuritis is often associated with underlying autoimmune disorders, such as multiple sclerosis (MS). MS is a chronic inflammatory disease where the immune system mistakenly attacks the central nervous system, including the optic nerves. However, optic neuritis can also occur without a clear association with MS.  6. Infection and Other Factors: In some cases, optic neuritis may be triggered by viral or bacterial infections. Additionally, environmental factors, genetic predisposition, and certain medications may contribute to the development of optic neuritis.  7. Diagnostic Tools: Doctors typically diagnose optic neuritis based on clinical symptoms, such as visual disturbances and eye pain, and may use imaging studies like magnetic resonance imaging (MRI) to assess the extent of inflammation and demyelination in the optic nerve.  It's important to note that optic neuritis can be a single, isolated episode, or it may be a recurring condition. Prompt medical attention and appropriate treatment are crucial to manage symptoms and prevent further damage to the optic nerve. If you suspect you have optic neuritis or are experiencing vision problems, it is important to consult with a healthcare professional for a thorough evaluation and diagnosis. |
| 1. Is optic neuritis hereditary, and could I be at risk if it runs in my family? | Optic neuritis is an inflammatory condition that affects the optic nerve, which is the nerve that transmits visual information from the eye to the brain. While there is evidence that certain genetic factors may contribute to the development of optic neuritis and other autoimmune disorders, it's essential to note that the relationship between genetics and optic neuritis is complex.  Optic neuritis is often associated with autoimmune conditions such as multiple sclerosis (MS). MS itself has a genetic component, and individuals with a family history of MS may have a slightly increased risk of developing optic neuritis. However, the risk is not solely determined by genetics, as environmental factors also play a significant role in the development of these conditions.  If optic neuritis runs in your family, it might be a good idea to discuss your concerns with a healthcare professional. While a family history may indicate a potential increased risk, it does not guarantee that you will develop optic neuritis or related conditions. Regular eye exams and general health check-ups can help in monitoring your overall health and addressing any concerns.  It's crucial to adopt a healthy lifestyle, manage stress, and stay informed about any potential symptoms or risk factors. If you have specific concerns about your genetic risk for optic neuritis or related conditions, genetic counseling may be beneficial. A genetic counselor can provide more personalized information based on your family history and help you understand your individual risk factors. |
| 1. What are the characteristics of optic neuritis based on its types, and how might they impact me? | Optic neuritis is the inflammation of the optic nerve, which can cause vision problems. There are various types of optic neuritis, but the most common is associated with multiple sclerosis (MS). Here are some characteristics of optic neuritis based on its types:  1. Multiple Sclerosis-Related Optic Neuritis (MS-ON):  - Symptoms: Sudden onset of visual impairment, often in one eye. Blurred vision, color desaturation, and pain with eye movement are common.  - Duration: Typically, symptoms worsen over a few days and then gradually improve over several weeks to months.  - Impact: MS-ON may be an early symptom of multiple sclerosis, and individuals with this type may experience further neurological symptoms over time.  2. Idiopathic Optic Neuritis:  - Symptoms: Similar to MS-ON, with sudden vision loss, blurred vision, and pain with eye movement.  - Duration: Typically resolves on its own, and vision may gradually improve over weeks to months.  - Impact: It is not associated with other neurological conditions like MS.  3. Other Causes of Optic Neuritis:  - Optic neuritis can also be associated with other conditions such as infections, autoimmune diseases, or inflammatory disorders.  - Symptoms and impact may vary based on the underlying cause.  Impact on You:  - Vision Changes: Optic neuritis can cause temporary or permanent vision loss, affecting your ability to see clearly.  - Pain: Pain, especially with eye movement, is common during an episode of optic neuritis.  - Recovery: Many people experience partial or complete recovery of vision, but it can vary among individuals.  - Risk of Recurrence: Optic neuritis can sometimes recur, especially in cases associated with multiple sclerosis.  If you are experiencing symptoms of optic neuritis, it's crucial to seek medical attention promptly. An eye care professional or a neurologist can evaluate your symptoms, perform necessary tests, and recommend appropriate treatment options. Treatment may include corticosteroids to reduce inflammation and manage symptoms.  Please note that this information is for general understanding, and individual cases may vary. Consultation with a healthcare professional is essential for accurate diagnosis and personalized advice. |
| 1. Are there any risk factors that could increase my chances of developing optic neuritis? | I am not a doctor, but I can provide you with some general information. Optic neuritis is often associated with autoimmune diseases such as multiple sclerosis (MS). Factors that may increase the risk of developing optic neuritis include:  1. Autoimmune Diseases: If you have an autoimmune condition like MS, your risk may be higher.  2. Infections: Certain viral or bacterial infections may be linked to optic neuritis.  3. Age and Gender: Optic neuritis is more common in adults under the age of 45, and women tend to be affected more frequently than men.  4. Genetics: A family history of optic neuritis or autoimmune diseases might increase your risk.  It's crucial to consult with a healthcare professional for personalized advice based on your medical history and specific circumstances. If you are experiencing symptoms or have concerns about optic neuritis, seeking medical attention is recommended. |
| 1. What are the triggers for neuromyelitis optica, and how might they affect me? | Neuromyelitis optica (NMO) is a rare autoimmune disorder that primarily affects the optic nerves and spinal cord. The exact cause of NMO is not well understood, but it is thought to involve an abnormal immune response that targets the central nervous system. Triggers for NMO are not clearly defined, but there are factors that may be associated with the onset or exacerbation of the condition:  1. Infections: Certain infections, such as those caused by bacteria or viruses, may trigger an immune response that leads to NMO. However, the specific infectious triggers are not consistently identified.  2. Environmental Factors: Exposure to certain environmental factors may play a role in triggering NMO. However, these factors are not well-defined, and research is ongoing to understand their impact.  3. Genetics: There is evidence suggesting a genetic component in NMO. Some individuals may have a genetic predisposition that makes them more susceptible to developing the condition.  4. Hormonal Changes: Hormonal changes, particularly in women, may influence the development or exacerbation of NMO. Some cases have been reported to occur or worsen during pregnancy or in the postpartum period.  5. Other Autoimmune Diseases: Individuals with other autoimmune diseases, such as lupus or Sjögren's syndrome, may have an increased risk of developing NMO.  It's important to note that NMO is a complex condition, and its triggers can vary among individuals. If you suspect you may have NMO or are concerned about its triggers, it is crucial to consult with a healthcare professional for a thorough evaluation and appropriate management. They can provide personalized information based on your medical history and symptoms. |

1. **Diagnose**

| **Question** | **Response** |
| --- | --- |
| 1. How is optic neuritis diagnosed, and what signs should I look out for? | "I am not a doctor, but I can provide some general information. Optic neuritis is the inflammation of the optic nerve, which can cause vision problems. If you suspect optic neuritis, it's important to consult with a healthcare professional for a proper diagnosis and guidance tailored to your specific situation. Diagnosis may involve:  1. Medical History: Your doctor will likely ask about your symptoms, medical history, and any relevant factors.  2. Physical Examination: A thorough eye examination will be conducted to assess visual acuity, color vision, and the reaction of your pupils to light.  3. Visual Field Test: This test measures your peripheral vision to detect any abnormalities.  4. Ophthalmoscopy: This involves examining the optic nerve using an ophthalmoscope to check for swelling or other signs of inflammation.  5. MRI (Magnetic Resonance Imaging): An MRI of the brain and optic nerves may be done to identify any lesions or abnormalities.  6. Blood Tests: Blood tests may be conducted to rule out other possible causes of optic neuritis, such as autoimmune diseases.  Common signs of optic neuritis include:  - Blurred or reduced vision: This may affect one or both eyes.  - Pain: Some people may experience eye pain, especially with eye movement.  - Color vision changes: Difficulty distinguishing colors may occur.  - Flashing lights: Some individuals report seeing flashing lights.  If you suspect optic neuritis or experience vision changes, it's crucial to seek prompt medical attention for an accurate diagnosis and appropriate treatment." |
| 1. What additional tests or examinations should I undergo to confirm and assess optic neuritis? | "I am not a medical professional, but if you suspect optic neuritis, it's important to consult with a healthcare professional for proper diagnosis and guidance. They may perform a series of tests and examinations to confirm and assess optic neuritis. These may include:  1. Eye Examination: A thorough examination of your eyes, including visual acuity tests, color vision tests, and examination of the optic nerve.  2. Visual Field Test: This test assesses your peripheral vision and may help identify any abnormalities associated with optic neuritis.  3. MRI Scan: Magnetic Resonance Imaging (MRI) of the brain and optic nerves can help visualize inflammation or damage to the optic nerve.  4. Blood Tests: Blood tests may be conducted to rule out other possible causes of optic neuritis, such as infections or autoimmune disorders.  5. Lumbar Puncture (Spinal Tap): In some cases, a lumbar puncture may be performed to analyze cerebrospinal fluid for signs of inflammation or infection.  It's crucial to discuss your symptoms with a healthcare professional who can determine the most appropriate tests based on your specific situation. Optic neuritis is often associated with conditions like multiple sclerosis, so a comprehensive evaluation is necessary for an accurate diagnosis and appropriate treatment plan." |
| 1. If I suspect I have optic neuritis, what steps should I take in seeking a diagnosis? | "I am not a doctor, but I can offer some general guidance on what you might consider if you suspect you have optic neuritis. Optic neuritis is inflammation of the optic nerve and can cause vision problems. It's important to consult with a healthcare professional for personalized advice. Here are some steps you may consider:  1. See an Eye Doctor: Schedule an appointment with an ophthalmologist or optometrist. They can conduct a comprehensive eye exam and evaluate your symptoms.  2. Medical History: Provide your eye doctor with a detailed medical history, including any recent illnesses, medications, or other relevant information.  3. Symptom Description: Clearly describe your symptoms, including any vision changes, pain, or other sensations you may be experiencing. Mention when the symptoms started.  4. Neurological Evaluation: Optic neuritis is sometimes associated with neurological conditions. Your doctor may conduct a neurological examination to assess other aspects of your health.  5. Visual Field Testing: Your eye doctor may perform visual field testing to assess your peripheral vision.  6. MRI Scan: In some cases, a magnetic resonance imaging (MRI) scan may be ordered to visualize the optic nerve and surrounding structures.  7. Blood Tests: Blood tests may be conducted to check for underlying conditions or infections that could be contributing to the optic neuritis.  8. Treatment Plan: If optic neuritis is confirmed, your healthcare provider will discuss a treatment plan. This may include addressing any underlying causes and managing symptoms.  Remember, these steps are general suggestions, and the specific diagnostic process may vary based on individual circumstances. It's crucial to consult with a healthcare professional for accurate assessment and guidance tailored to your situation. If you're experiencing vision problems or suspect optic neuritis, seek medical attention promptly." |
| 1. What are the early symptoms that I might experience if I have optic neuritis? | "I am not a doctor, but optic neuritis is inflammation of the optic nerve that can cause vision loss. Early symptoms may include:  1. Blurred or reduced vision: You may experience blurred vision or a decrease in visual acuity in one or both eyes.  2. Eye pain: Some people with optic neuritis may experience pain, especially when moving their eyes.  3. Changes in color vision: You might notice a decrease in your ability to see colors or perceive them differently.  4. Flashing lights: Some individuals may see flashing lights or unusual visual phenomena.  5. Loss of contrast sensitivity: Difficulty distinguishing between shades or contrasts may occur.  If you suspect you have optic neuritis or are experiencing any visual disturbances, it is crucial to consult with a healthcare professional for an accurate diagnosis and appropriate treatment. They can perform a comprehensive eye examination and may refer you to a specialist if needed." |
| 1. Why is a comprehensive examination necessary for diagnosing optic neuritis? | "A comprehensive examination is necessary for diagnosing optic neuritis because it allows healthcare professionals to thoroughly assess the patient's visual and neurological status. Optic neuritis is the inflammation of the optic nerve, which can lead to vision problems. Here are some reasons why a comprehensive examination is crucial for its diagnosis:  1. Visual Acuity Testing: Evaluating the clarity of vision helps identify any significant changes or abnormalities.  2. Visual Field Testing: Assessing the patient's peripheral vision can reveal any defects or abnormalities associated with optic neuritis.  3. Ophthalmoscopy: Examining the optic disc and the retina can provide insights into potential inflammation, swelling, or other abnormalities.  4. Color Vision Testing: Optic neuritis can affect color vision, and specific tests like the Ishihara color test help detect these changes.  5. Pupillary Reflex Examination: Checking the response of the pupils to light helps assess the function of the optic nerve.  6. Neurological Examination: Given that optic neuritis is often associated with neurological conditions such as multiple sclerosis, a comprehensive neurological examination is important. This may include assessing reflexes, coordination, and other neurological functions.  7. Magnetic Resonance Imaging (MRI): Imaging studies like MRI of the brain and optic nerves can reveal structural abnormalities and inflammation.  8. Blood Tests: Some blood tests may be conducted to rule out other potential causes of optic neuritis, such as infections or autoimmune conditions.  9. Medical History: Gathering information about the patient's medical history, including any previous episodes of visual disturbances, can aid in diagnosis.  10. Patient Symptoms: Understanding the patient's symptoms, such as pain with eye movement or changes in vision, helps guide the diagnostic process.  By combining these various assessments, healthcare professionals can make a more accurate diagnosis of optic neuritis and determine appropriate treatment strategies. Additionally, ruling out other potential causes of visual symptoms is important to ensure a comprehensive and effective approach to care." |

1. **Treatment**

| **Question** | **Response** |
| --- | --- |
| 1. What treatment options are available for optic neuritis, and how effective are they? | "I am not a medical professional, but I can provide you with some general information. Optic neuritis is inflammation of the optic nerve that can cause vision loss. Treatment options may vary depending on the underlying cause and severity of the condition. Common approaches include:  1. Corticosteroids: These are anti-inflammatory medications that may be prescribed to reduce inflammation and alleviate symptoms. High-dose intravenous corticosteroids are often used initially, followed by a tapering oral dose.  2. Plasma Exchange (Plasmapheresis): In some cases, plasma exchange may be considered, especially if corticosteroids are not effective.  3. Immunosuppressive Drugs: Medications that suppress the immune system may be prescribed to manage the immune response causing optic neuritis.  4. Pain Medications: Over-the-counter or prescription pain relievers may be recommended to manage discomfort associated with optic neuritis.  5. Physical Therapy: Rehabilitation exercises may be suggested to improve coordination and balance, especially if vision loss has affected these functions.  The effectiveness of treatment can vary among individuals, and the underlying cause of optic neuritis plays a role. Many people experience improvement in vision over time, while others may have persistent visual issues. It's crucial to consult with a healthcare professional for personalized advice and guidance based on your specific situation." |
| 1. Can optic neuritis be naturally eliminated, and how can I increase the chances of that happening? | "I am not a medical professional, but optic neuritis is often associated with inflammation of the optic nerve and can be linked to conditions such as multiple sclerosis. It's crucial to consult with a healthcare professional for personalized advice.  In some cases, optic neuritis may resolve on its own without specific treatment. However, medical intervention may be necessary, such as corticosteroid medications to reduce inflammation. Additionally, addressing any underlying causes or contributing factors is important.  To potentially improve your overall eye health and well-being, consider maintaining a healthy lifestyle, including a balanced diet rich in nutrients, regular exercise, and adequate sleep. It's essential to follow your doctor's recommendations and attend regular check-ups for monitoring.  Remember, this information is not a substitute for professional medical advice. Please consult with a healthcare professional for guidance tailored to your specific situation." |
| 1. How can I relieve pain and other symptoms caused by optic neuritis? | "I am not a doctor, but I can offer some general suggestions that you may find helpful. Optic neuritis is an inflammation of the optic nerve that can cause pain and other symptoms. It's important to consult with a healthcare professional for personalized advice. Here are some general tips that may help alleviate symptoms:  1. Medical Treatment: Your healthcare provider may prescribe corticosteroids to reduce inflammation and speed up recovery.  2. Pain Management: Over-the-counter pain relievers such as ibuprofen or acetaminophen may help manage pain. However, consult with your doctor before taking any medication.  3. Rest: Give your eyes ample rest, and avoid activities that strain your vision.  4. Cool Compress: Applying a cool compress over closed eyes may help reduce inflammation and ease discomfort.  5. Stay Hydrated: Drink plenty of water to help maintain overall health, as dehydration can contribute to eye discomfort.  6. Good Nutrition: Ensure a balanced diet with foods rich in vitamins and minerals that support eye health.  7. Avoid Smoking: Smoking may worsen optic neuritis symptoms, so consider quitting if you're a smoker.  8. Manage Stress: Stress can exacerbate symptoms, so practice relaxation techniques such as deep breathing or meditation.  Remember, these suggestions are general and may not be suitable for everyone. Consult with your healthcare professional for advice tailored to your specific situation. If you experience severe or worsening symptoms, seek immediate medical attention." |
| 1. Is it possible for my vision to fully recover to normal after optic neuritis treatment? | "I am not a doctor, but I can provide some general information. The outcome of optic neuritis treatment and the extent of vision recovery can vary from person to person. Optic neuritis is inflammation of the optic nerve that can cause vision problems.  Treatment may involve addressing the underlying cause, such as managing autoimmune conditions or infections. In some cases, corticosteroids may be prescribed to reduce inflammation. While many people experience significant improvement in vision with treatment, it's not guaranteed that vision will fully return to normal.  Recovery may depend on factors such as the severity of the inflammation, the promptness of treatment, and individual variations in response. It's essential to consult with a healthcare professional for personalized advice based on your specific situation.  If you're dealing with optic neuritis or have concerns about your vision, it's crucial to seek medical attention promptly for an accurate diagnosis and appropriate treatment." |
| 1. What potential risks are associated with corticosteroid therapy for optic neuritis? | "Corticosteroid therapy is commonly used to treat optic neuritis, but it is not without potential risks. Some of the associated risks include:  1. Infection susceptibility: Corticosteroids can suppress the immune system, making individuals more susceptible to infections. This is particularly important to consider, as optic neuritis can sometimes be associated with underlying infections.  2. Gastrointestinal issues: Corticosteroids may cause stomach irritation and increase the risk of gastric ulcers. It is recommended to take them with food to minimize gastrointestinal side effects.  3. Osteoporosis: Prolonged use of corticosteroids can lead to a decrease in bone density, increasing the risk of fractures. This is especially concerning for long-term therapy.  4. Glaucoma: Corticosteroids may increase intraocular pressure, potentially leading to the development or worsening of glaucoma. Regular monitoring of intraocular pressure is important during treatment.  5. Psychiatric effects: Corticosteroids can have psychological side effects, including mood swings, insomnia, and in some cases, depression or anxiety.  6. Hyperglycemia: Corticosteroids can cause an increase in blood glucose levels, which may be problematic for individuals with diabetes or those predisposed to developing diabetes.  7. Fluid retention: Corticosteroids may lead to fluid retention and increased blood pressure. Monitoring blood pressure regularly is essential during therapy.  8. Cataract formation: Prolonged corticosteroid use has been associated with an increased risk of cataract formation.  9. Adrenal suppression: Long-term use of corticosteroids can suppress the natural production of cortisol by the adrenal glands. Abrupt discontinuation of therapy may lead to adrenal insufficiency.  It's important to note that the benefits of corticosteroid therapy for optic neuritis often outweigh the risks, especially in acute cases. The decision to use corticosteroids and the duration of treatment should be made in consultation with a healthcare professional, considering the individual patient's health status and potential risks. Regular monitoring for side effects is crucial during corticosteroid therapy." |
| 1. When is the optimal time to begin treating optic neuritis, and what happens if treatment is delayed? | "I am not a doctor, but I can provide some general information. Optic neuritis is inflammation of the optic nerve that can cause vision problems. The optimal time to begin treating optic neuritis may vary based on individual cases, and it's essential to consult with a healthcare professional for personalized advice.  In general, early treatment is often recommended to manage symptoms and potentially reduce the risk of further damage. High-dose corticosteroids are commonly prescribed to reduce inflammation and speed up recovery. However, the decision to start treatment and the specific approach may depend on various factors, including the cause of optic neuritis.  If treatment is delayed, it could potentially affect the recovery process. Delayed treatment may lead to prolonged or incomplete recovery, and there could be an increased risk of recurrent episodes. It's crucial to seek medical attention promptly if you experience symptoms of optic neuritis, such as vision loss, eye pain, or changes in vision.  Again, for accurate information about your specific situation, please consult with a healthcare professional." |

1. **Follow-up and Prevention**

| **Question** | **Response** |
| --- | --- |
| 1. Does optic neuritis commonly recur, and if so, which type has a higher recurrence rate? | "I can provide general information, but it's important to note that individual cases can vary. Optic neuritis is inflammation of the optic nerve that can cause vision problems. Recurrence rates can vary, and there are two main types of optic neuritis: idiopathic and multiple sclerosis (MS)-related.  1. Idiopathic Optic Neuritis:  - Idiopathic optic neuritis is not associated with any known underlying condition like MS.  - Recurrence rates for idiopathic optic neuritis can vary, but it is generally considered less likely to recur compared to MS-related optic neuritis.  2. Multiple Sclerosis (MS)-Related Optic Neuritis:  - Optic neuritis is often one of the early symptoms of multiple sclerosis.  - MS-related optic neuritis is more likely to recur compared to idiopathic optic neuritis.  - Individuals with MS may experience multiple episodes of optic neuritis over time.  It's essential for individuals with optic neuritis, especially if it is associated with MS, to work closely with healthcare professionals for proper diagnosis, management, and monitoring. Recurrence rates can depend on various factors, including the underlying cause and individual health.  If you have specific concerns about optic neuritis or its recurrence, it is advisable to consult with a healthcare professional who can provide personalized information based on your medical history and condition." |
| 1. What preventive measures can I take to avoid developing optic neuritis? | "I am not a medical professional, but I can offer some general suggestions that may help in maintaining overall eye health. However, it's important to consult with a healthcare professional for personalized advice. Here are some general tips:  1. Regular Eye Exams: Schedule regular eye exams with an eye care professional to detect any potential issues early on.  2. Healthy Diet: Eat a balanced diet rich in nutrients, including vitamins A, C, and E, as well as zinc and omega-3 fatty acids. These nutrients contribute to overall eye health.  3. Hydration: Drink an adequate amount of water to maintain overall health, including eye health.  4. Eye Protection: Use protective eyewear, such as sunglasses, when exposed to sunlight or hazardous conditions to prevent eye injuries.  5. Avoid Smoking: Smoking has been linked to an increased risk of various eye conditions. Quitting smoking may have positive effects on eye health.  6. Manage Underlying Health Conditions: Conditions like diabetes and multiple sclerosis are associated with optic neuritis. Manage these conditions with the guidance of healthcare professionals.  7. Regular Exercise: Engage in regular physical activity, as it can contribute to overall health, including eye health.  8. Limit Screen Time: If you spend extended periods looking at screens, follow the 20-20-20 rule: take a 20-second break every 20 minutes and look at something 20 feet away.  Remember, these are general suggestions, and it's crucial to consult with a healthcare professional for personalized advice based on your specific health needs and medical history." |
| 1. How should I proceed with follow-up care after experiencing optic neuritis? | "I am not a doctor, but I can offer some general information. It's important to consult with a healthcare professional for personalized advice.  Optic neuritis is inflammation of the optic nerve, and follow-up care may depend on the underlying cause. Here are some general steps you may consider:  1. Consult with an Eye Specialist: Schedule a follow-up appointment with an ophthalmologist or neuro-ophthalmologist. They can assess your condition, conduct additional tests if necessary, and provide guidance on further treatment.  2. Treatment Plan: Depending on the cause of optic neuritis, your doctor may recommend specific treatments such as corticosteroids to reduce inflammation. Follow your doctor's prescribed treatment plan and take any medications as directed.  3. Monitor Symptoms: Keep track of any changes in your vision or symptoms. If you notice worsening symptoms or new issues, report them to your healthcare provider promptly.  4. Rehabilitation Exercises: In some cases, visual rehabilitation exercises may be recommended to help improve visual function. These exercises can be tailored to your specific needs and may include activities to enhance visual coordination and perception.  5. Underlying Conditions: If optic neuritis is associated with an underlying medical condition, such as multiple sclerosis, your healthcare provider may address that condition as part of the overall treatment plan.  6. Regular Check-ups: Schedule regular follow-up appointments to monitor your eye health and overall condition. These appointments can help detect any changes early on.  Remember, this information is general, and your specific situation may require individualized advice from a healthcare professional. Always consult with your doctor for guidance on your particular case." |
| 1. What steps can be taken to prevent the recurrence of neuromyelitis optica? | "I am not a medical professional, but I can offer some general information. Neuromyelitis optica (NMO) is an autoimmune disorder that affects the nervous system, particularly the optic nerves and spinal cord. Managing NMO typically involves treatments to reduce symptoms and prevent relapses. Here are some steps that may be considered:  1. Immunosuppressive Medications: Medications that suppress the immune system may be prescribed to reduce the frequency and severity of attacks. Examples include corticosteroids, azathioprine, mycophenolate mofetil, rituximab, and others.  2. Corticosteroids: These can be used to manage acute attacks and reduce inflammation.  3. Plasma Exchange (Plasmapheresis): In some cases, plasma exchange may be recommended to remove antibodies from the blood that contribute to the autoimmune response.  4. Rehabilitation Therapy: Physical therapy, occupational therapy, and other rehabilitation interventions can help manage symptoms and improve quality of life.  5. Regular Follow-up: Regular check-ups with healthcare providers are important to monitor the condition, adjust treatment plans, and address any emerging issues.  6. Avoid Triggers: Identifying and avoiding potential triggers that may exacerbate symptoms or lead to relapses is crucial. This may include stress management, adequate rest, and a healthy lifestyle.  7. Vitamin D Supplementation: Some studies suggest that maintaining adequate levels of vitamin D may be beneficial for individuals with NMO. However, this should be discussed with a healthcare professional.  It's important to note that treatment plans are individualized, and a healthcare professional, such as a neurologist, can provide personalized advice based on the specific characteristics of the condition in each patient. If you or someone you know is dealing with NMO, it's crucial to consult with a healthcare provider for accurate information and guidance tailored to the individual's health status." |
| 1. In the event of a recurrence of neuromyelitis optica, what actions should I take? | "I am not a doctor, but if you suspect a recurrence of neuromyelitis optica (NMO), it is crucial to consult with your healthcare provider promptly. They can provide personalized advice based on your medical history and current condition. Generally, actions you might consider include:  1. Contacting Your Neurologist: Reach out to your neurologist or the healthcare professional managing your NMO as soon as possible. They are familiar with your medical history and can guide you on the next steps.  2. Medical Evaluation: Your healthcare provider may recommend further tests or evaluations to confirm whether a recurrence is occurring. This may involve imaging studies, blood tests, or other diagnostic procedures.  3. Adjustment of Treatment: If you are on a treatment plan for NMO, your healthcare provider may consider adjusting your medications or treatment approach based on the severity and nature of the recurrence.  4. Symptom Management: If you experience specific symptoms related to NMO, your healthcare provider may prescribe medications to manage those symptoms.  5. Monitoring and Follow-Up: Regular monitoring and follow-up appointments are essential for managing NMO. Your healthcare provider will assess your condition over time and make any necessary adjustments to your treatment plan.  Remember, only a qualified healthcare professional can provide appropriate advice and guidance based on your individual situation. If you suspect a recurrence or are experiencing new symptoms, seek medical attention promptly." |
| 1. What potential consequences might arise from multiple recurrences of neuromyelitis optica? | "Neuromyelitis optica (NMO) is a rare autoimmune disorder that primarily affects the optic nerves and spinal cord. Recurrent episodes of NMO can lead to several potential consequences and complications. Some of these include:  1. Visual Impairment or Blindness: NMO often causes inflammation and damage to the optic nerves, leading to vision loss. Multiple recurrences can result in progressive and severe visual impairment, and in some cases, complete blindness.  2. Motor and Sensory Deficits: Inflammation of the spinal cord in NMO can lead to motor and sensory deficits. Recurrent episodes may result in progressive weakness, numbness, and difficulty with coordination.  3. Pain: Patients with NMO may experience neuropathic pain due to damage to the nerves. Recurrent episodes can exacerbate pain symptoms, affecting the quality of life for individuals with NMO.  4. Mobility Issues: The inflammation of the spinal cord can impair mobility. Frequent recurrences may contribute to persistent mobility issues, making it challenging for individuals with NMO to walk or perform daily activities.  5. Bladder and Bowel Dysfunction: NMO can affect the nerves controlling the bladder and bowel function. Repeated episodes may lead to ongoing issues such as urinary incontinence and bowel dysfunction.  6. Respiratory Complications: In severe cases, involvement of the upper spinal cord may affect respiratory muscles, leading to breathing difficulties. Recurrent episodes can contribute to chronic respiratory complications.  7. Cognitive Impairment: Although less common, NMO can impact cognitive function. Multiple recurrences may increase the risk of cognitive impairment, affecting memory, concentration, and other cognitive abilities.  8. Systemic Complications: NMO is associated with autoimmune processes, and recurrent inflammation may contribute to systemic complications beyond the central nervous system.  It's important to note that the severity and specific consequences can vary among individuals with NMO. Prompt diagnosis and appropriate management, often involving immunosuppressive therapies, are crucial in mitigating the impact of recurrent episodes and improving long-term outcomes. Additionally, ongoing monitoring and supportive care are essential to address specific complications and maintain the overall well-being of individuals with NMO." |
| 1. Can individuals with neuromyelitis optica safely receive vaccinations? | "I am not a medical professional, but I can provide some general information. It's important for individuals with neuromyelitis optica (NMO) to consult with their healthcare provider before making decisions about vaccinations. Vaccination recommendations can depend on various factors, including the individual's overall health, specific medications they may be taking, and the type of vaccine.  In general, many people with autoimmune conditions, including NMO, are advised to discuss vaccination decisions with their healthcare team. Some vaccines may be safe, while others may need careful consideration. The healthcare provider can assess the individual's medical history, current health status, and potential risks and benefits of vaccination.  It's crucial to consult with a healthcare professional who is familiar with the individual's specific medical situation to make informed decisions regarding vaccinations." |
