# Supplementary material for: Evaluation and comparison of large language models’ responses to questions related optic neuritis
Source: Front Med (Lausanne). 2025 Jun 25;12:1516442. doi: 10.3389/fmed.2025.1516442 (PMC12238082; doi:10.3389/fmed.2025.1516442)
Supplement: Supplementary file 4 [file Table_4.docx]

**Table S4**

**ChatGPT-4.0**

1. **General**

| **Question** | **Response** |
| --- | --- |
| 1. What is optic neuritis, and how will it impact my vision? | Optic neuritis is an inflammation of the optic nerve, which is crucial for vision as it transmits visual information from the retina to the brain. It can be caused by various factors, including infections, immune-mediated diseases like multiple sclerosis, and other systemic conditions.  The impact on vision varies, but common symptoms include:  1. Blurred or reduced vision in one eye.  2. Pain, often worsening with eye movement.  3. Loss of color vision or colors appearing less vivid.  4. A dark spot in the center of your vision (central scotoma).  The extent and duration of these symptoms can vary. Some people experience a significant reduction in vision, while others may have milder effects. The prognosis depends on the underlying cause, and in many cases, vision improves over time, especially with treatment. It's important to seek medical advice for a proper diagnosis and treatment plan. |
| 1. Could you elaborate on the specific process leading to the formation of optic neuritis? | Optic neuritis is an inflammation of the optic nerve, which is crucial for vision. The process leading to the formation of optic neuritis involves several steps:  1. Immune System Activation: Optic neuritis typically occurs when the immune system mistakenly targets the optic nerve, leading to inflammation. This can be part of an autoimmune response where the body's defense system attacks its own tissues.  2. Inflammation: The immune response leads to inflammation of the optic nerve. This inflammation can cause swelling and damage to the nerve fibers (axons) and their myelin sheath, which is the protective covering around nerve fibers. In diseases like multiple sclerosis (MS), the myelin sheath is specifically targeted.  3. Demyelination: The process of myelin sheath damage is known as demyelination. Myelin is essential for the proper and fast conduction of electrical signals along the nerve fibers. When demyelination occurs, the transmission of visual information from the eye to the brain is disrupted or slowed.  4. Symptoms Development: As a result of this disruption, symptoms of optic neuritis develop. These can include blurred vision, loss of color vision, pain with eye movement, and in some cases, complete vision loss in the affected eye.  5. Recovery or Progression: The course of optic neuritis can vary. Some individuals may experience a partial or complete recovery of vision, often within weeks to months, as the inflammation subsides and the nerve repairs itself. However, in conditions like MS, optic neuritis may recur or lead to further neurological symptoms.  6. Underlying Causes: While optic neuritis can occur on its own, it is often associated with other conditions, such as multiple sclerosis, neuromyelitis optica, infections, or other autoimmune disorders. Identifying and treating any underlying condition is crucial in managing optic neuritis.  The exact cause of the autoimmune response leading to optic neuritis is not entirely understood and is likely multifactorial, involving genetic predisposition, environmental factors, and possibly infections. Diagnosis and treatment typically involve a combination of clinical evaluation, imaging (like MRI), and sometimes steroid therapy to reduce inflammation and aid recovery. |
| 1. Is optic neuritis hereditary, and could I be at risk if it runs in my family? | Optic neuritis, an inflammation of the optic nerve that can cause vision loss, is generally not considered a hereditary condition. However, it can be associated with certain autoimmune disorders, like multiple sclerosis (MS), which may have a genetic component.  If optic neuritis runs in your family, it may be due to an underlying condition that has a hereditary aspect. It's important to understand that having a family history of optic neuritis or related conditions doesn't necessarily mean you will develop it, but it may increase your risk compared to the general population.  It's advisable to consult with a healthcare professional for a personalized assessment of your risk. They can provide more information based on your family's medical history and any symptoms you might be experiencing. Regular eye exams and monitoring for symptoms of optic neuritis, such as vision loss, pain with eye movement, or changes in color vision, are also important if you have concerns about this condition. |
| 1. What are the characteristics of optic neuritis based on its types, and how might they impact me? | Optic neuritis is an inflammation of the optic nerve that can lead to a temporary or permanent reduction in vision. The characteristics of optic neuritis vary depending on the type and underlying cause. Here are some common types and their characteristics:  1. Idiopathic Optic Neuritis: This is the most common form, often associated with demyelinating diseases like multiple sclerosis (MS). Characteristics include:  - Sudden loss of vision in one eye, usually over hours or days.  - Pain that worsens with eye movement.  - A central vision loss or a field defect.  - Loss of color vision or washed-out colors.  - Recovery of vision typically begins within a few weeks, but some level of vision loss can be permanent.  2. Neuromyelitis Optica (NMO) Spectrum Optic Neuritis: NMO is a separate disease from MS, and optic neuritis in NMO can be more severe. Characteristics include:  - Severe vision loss, potentially more profound than in idiopathic optic neuritis.  - Pain with eye movement.  - Possible recurrent episodes, which can lead to cumulative damage.  3. Chronic Relapsing Inflammatory Optic Neuropathy (CRION): This is a recurrent form of optic neuritis that responds to steroids. Characteristics include:  - Recurrent episodes of optic neuritis.  - Vision loss with each episode, which improves with steroid treatment.  - Dependency on steroids to prevent relapses.  4. Optic Neuritis Associated with Systemic Conditions: Conditions like lupus, sarcoidosis, or infections can cause optic neuritis. Characteristics depend on the underlying condition but can include:  - Variable vision loss.  - Pain with eye movement.  - Possible other symptoms related to the systemic condition.  Impact on an individual can vary significantly based on the type and severity of optic neuritis. Some potential impacts include:  - Visual Impairment: The degree of vision loss can range from mild to severe, and while some people recover fully, others may have permanent vision loss.  - Pain: Eye pain, especially with movement, can be a significant symptom.  - Color Vision Changes: Reduction in color vision or dullness of colors is common.  - Relapses: In conditions like NMO or CRION, recurrent episodes can lead to cumulative damage and worsening vision over time.  - Associated Conditions: If optic neuritis is part of a broader condition like MS or lupus, other symptoms related to these conditions can also impact overall health and well-being.  It's important to consult with a healthcare professional for an accurate diagnosis and appropriate management based on the specific type of optic neuritis and individual health circumstances. |
| 1. Are there any risk factors that could increase my chances of developing optic neuritis? | Yes, there are several risk factors that can increase the likelihood of developing optic neuritis. Optic neuritis is an inflammation of the optic nerve that can cause vision loss. The risk factors include:  1. Autoimmune Diseases: Conditions like multiple sclerosis (MS) and neuromyelitis optica increase the risk of optic neuritis. In MS, the immune system attacks the protective sheath (myelin) covering nerve fibers, which can affect the optic nerve.  2. Age and Gender: Optic neuritis most commonly affects young adults, and women are at higher risk than men.  3. Genetic Factors: Some genetic predispositions may increase the risk, though specific genes haven't been definitively linked to optic neuritis.  4. Infections: Certain viral and bacterial infections, such as Lyme disease, syphilis, measles, mumps, and herpes viruses, can trigger or be associated with optic neuritis.  5. Other Medical Conditions: Conditions that affect the immune system, like lupus or sarcoidosis, can also increase the risk.  6. Certain Medications: Some drugs, especially those that affect the immune system, can increase the risk of optic neuritis.  7. Environmental Factors: Smoking and possibly other environmental factors might contribute to the risk.  It's important to note that having one or more risk factors doesn't mean you will definitely develop optic neuritis. Also, people without these risk factors can still develop the condition. If you have concerns about optic neuritis, especially if you have symptoms like vision loss, pain with eye movement, or other related symptoms, it's important to consult a healthcare professional for an accurate diagnosis and appropriate management. |
| 1. What are the triggers for neuromyelitis optica, and how might they affect me? | Neuromyelitis optica (NMO), also known as Devic's disease, is a rare neurological condition that primarily affects the eyes and spinal cord. The exact causes of NMO are not fully understood, but it is believed to be an autoimmune disorder. In people with NMO, the immune system mistakenly attacks healthy cells in the central nervous system, particularly targeting the optic nerves and spinal cord.  Triggers for NMO can vary among individuals, and not everyone with NMO will have identifiable triggers. However, some potential triggers or factors that might exacerbate the condition include:  1. Infections: Certain viral or bacterial infections can sometimes trigger a relapse or worsen symptoms. These infections might stimulate the immune system in a way that leads to an autoimmune response against the central nervous system.  2. Stress: Physical or emotional stress may contribute to the worsening of symptoms or trigger a relapse. Stress can affect the immune system and potentially exacerbate autoimmune conditions.  3. Fatigue: Overexertion and lack of rest might trigger symptoms or make them worse.  4. Heat and Temperature Changes: Exposure to heat, such as from hot weather, hot baths, or fever, can worsen symptoms or trigger a relapse. This is known as Uhthoff's phenomenon, which is common in demyelinating diseases like NMO and multiple sclerosis.  5. Hormonal Changes: Some individuals might experience changes in symptoms related to hormonal fluctuations, such as those occurring during menstruation, pregnancy, or menopause.  6. Other Autoimmune Diseases: Having another autoimmune disorder may increase the risk or affect the course of NMO.  7. Certain Medications: Some medications might trigger or worsen symptoms, especially those affecting the immune system.  How these triggers affect you specifically can vary based on individual factors such as your overall health, the severity of your NMO, and your treatment regimen. It's important to work closely with your healthcare provider to manage your condition effectively. They can help you identify potential triggers and develop strategies to avoid them, as well as provide treatments to manage symptoms and reduce the frequency of relapses. |

1. **Diagnose**

| **Question** | **Response** |
| --- | --- |
| 1. How is optic neuritis diagnosed, and what signs should I look out for? | "Optic neuritis is an inflammation of the optic nerve that can cause a sudden decrease in vision. The diagnosis is typically made based on a combination of clinical symptoms, a comprehensive eye examination, and imaging tests. Here are the key aspects:  1. Clinical Symptoms: The most common symptom of optic neuritis is a sudden loss of vision in one eye, which can occur over a few hours to a few days. This loss of vision is often accompanied by pain, especially when moving the eye. Other symptoms might include changes in color vision (colors appear dull) and a flickering effect in the vision.  2. Comprehensive Eye Examination: An eye doctor (ophthalmologist or optometrist) will perform a thorough examination. This may include testing visual acuity, color vision, and a detailed examination of the optic nerve using an instrument called an ophthalmoscope. A relative afferent pupillary defect (RAPD) is often present, which is a specific finding on the pupil examination when light is shone in the eyes.  3. Visual Field Testing: This test measures peripheral vision and can identify specific patterns of vision loss associated with optic neuritis.  4. Optical Coherence Tomography (OCT): This non-invasive imaging test provides detailed images of the retina and optic nerve and can show thinning of the nerve fiber layer in cases of optic neuritis.  5. Magnetic Resonance Imaging (MRI): An MRI of the brain and orbits (eye sockets) with gadolinium contrast is often performed to view the optic nerves and identify any lesions that might indicate multiple sclerosis (MS) or other neurological conditions.  6. Blood Tests: Sometimes, blood tests are conducted to rule out other conditions that can mimic optic neuritis, such as infections or autoimmune diseases.  If you suspect that you or someone else is experiencing symptoms of optic neuritis, it's important to seek medical attention promptly. Early diagnosis and treatment are crucial for the best possible outcome and to address any underlying conditions that may be associated with optic neuritis, such as multiple sclerosis." |
| 1. What additional tests or examinations should I undergo to confirm and assess optic neuritis? | "If you suspect you have optic neuritis, it's essential to consult a healthcare professional for a proper diagnosis and treatment. A neurologist or ophthalmologist typically performs several tests and examinations to confirm optic neuritis and assess its severity. Here are some common tests and procedures:  1. Eye Examination: A thorough examination of the eye, including testing of visual acuity, color vision, and visual field, as well as a fundoscopic exam to look at the optic nerve.  2. Pupillary Light Reflex Test: This test evaluates the reaction of your pupils to light, which can be affected in optic neuritis.  3. Optical Coherence Tomography (OCT): A non-invasive imaging test that provides detailed images of the retina and can measure the thickness of the retinal nerve fiber layer, which may be reduced in optic neuritis.  4. Visual Evoked Potentials (VEP): This test measures the electrical response of the brain to visual stimuli. Delays in response can indicate optic nerve damage.  5. Magnetic Resonance Imaging (MRI): An MRI of the brain and orbits can show inflammation and lesions on the optic nerve and can also help in diagnosing multiple sclerosis, a common cause of optic neuritis.  6. Blood Tests: While not specific for optic neuritis, blood tests can help rule out other conditions that may mimic optic neuritis, such as infections or autoimmune diseases.  7. Lumbar Puncture: In some cases, a spinal tap may be performed to analyze the cerebrospinal fluid, particularly if multiple sclerosis is suspected.  Remember, only a healthcare professional can recommend the appropriate tests based on your specific condition and symptoms. It's crucial to follow their guidance for accurate diagnosis and effective treatment." |
| 1. If I suspect I have optic neuritis, what steps should I take in seeking a diagnosis? | "If you suspect you have optic neuritis, it's important to follow these steps to seek a diagnosis:  1. Consult a Healthcare Professional: Make an appointment with your primary care physician or an eye specialist (ophthalmologist or optometrist) as soon as possible. Explain your symptoms in detail.  2. Detailed Eye Examination: An eye specialist will conduct a thorough examination of your eyes. This may include testing your visual acuity, color vision, pupil reactions, and looking at the optic nerve through an ophthalmoscope.  3. Medical History: Be prepared to discuss your medical history, including any other symptoms you might be experiencing and any family history of similar conditions.  4. Referral to a Neurologist: If your eye specialist suspects optic neuritis, you may be referred to a neurologist for further evaluation, as optic neuritis can be associated with neurological conditions like multiple sclerosis (MS).  5. Additional Tests: The neurologist may order additional tests, such as an MRI (Magnetic Resonance Imaging) to view the optic nerve and brain, and Visual Evoked Potentials (VEP) to test the electrical activity in your visual system.  6. Discuss Treatment Options: Treatment for optic neuritis might include corticosteroids to reduce inflammation. Your healthcare provider will discuss the best treatment options for your specific situation.  7. Regular Follow-Up: Regular follow-up appointments are crucial to monitor the condition and any changes in your vision.  8. Consider Lifestyle Modifications: Depending on the underlying cause, lifestyle changes such as managing stress, improving diet, and avoiding smoking might be recommended.  Remember, early diagnosis and treatment are key to managing optic neuritis effectively. Do not delay seeking medical advice if you have concerns about your vision." |
| 1. What are the early symptoms that I might experience if I have optic neuritis? | "Optic neuritis is an inflammation of the optic nerve that can cause a range of symptoms. Early symptoms of optic neuritis can include:  1. Vision Loss: This is often the first and most noticeable symptom. It can be mild or severe and usually affects one eye. The loss of vision may develop over a few days and can vary from a small area of blurred vision to complete blindness in the affected eye.  2. Pain: Many people experience pain with eye movement. The pain can be mild to severe and typically precedes the loss of vision.  3. Changes in Color Vision: You might notice that colors appear less bright or vivid, particularly reds, which may appear washed out or faded.  4. Flashing Lights: Some people experience flashing or flickering lights, particularly when moving their eyes.  5. Visual Field Loss: This refers to a loss of part of the usual field of view, which can present as a dark spot or area in your vision.  6. Reduced Contrast Sensitivity: This means difficulty in distinguishing between shades of gray.  Remember, these symptoms are not exclusive to optic neuritis and can be caused by other eye or neurological conditions. If you experience these symptoms, it's important to see a healthcare professional for a proper diagnosis and treatment. Optic neuritis can be associated with other conditions, such as multiple sclerosis, and early treatment can be important for overall health and vision preservation." |
| 1. Why is a comprehensive examination necessary for diagnosing optic neuritis? | "A comprehensive examination is necessary for diagnosing optic neuritis because it's a condition that can have various causes and manifestations, and it's important to accurately determine the underlying cause and rule out other possible conditions. Here's why different aspects of a comprehensive examination are important:  1. Medical History: Understanding the patient's medical history helps in identifying any risk factors or related conditions that might be associated with optic neuritis, such as multiple sclerosis, infections, or autoimmune disorders.  2. Visual Symptoms Assessment: Since optic neuritis primarily affects vision, a detailed assessment of visual symptoms like loss of vision, pain with eye movement, and changes in color vision is crucial.  3. Ophthalmologic Examination: This includes testing for visual acuity, color vision, and visual field. A fundoscopic exam, where the back of the eye is examined, can reveal swelling of the optic disc or other abnormalities.  4. Pupillary Light Reflex Test: This test checks the reaction of the pupils to light. An abnormal response can be a sign of optic nerve damage.  5. Neurological Examination: Optic neuritis can be associated with neurological disorders like multiple sclerosis, so a neurological exam may be conducted to check for other signs of neurological impairment.  6. Imaging Tests: MRI (Magnetic Resonance Imaging) of the brain and orbits can help in identifying inflammation of the optic nerve and ruling out other conditions like tumors or other causes of optic nerve damage.  7. Blood Tests: These can help in identifying underlying systemic conditions like infections or autoimmune diseases that might cause or contribute to optic neuritis.  8. Lumbar Puncture: In some cases, a spinal tap may be done to analyze the cerebrospinal fluid for abnormalities that are associated with certain conditions like multiple sclerosis.  A comprehensive examination ensures a correct diagnosis and appropriate treatment plan, considering optic neuritis can be a symptom of a broader health issue and its management may vary depending on the underlying cause." |

1. **Treatment**

| **Question** | **Response** |
| --- | --- |
| 1. What treatment options are available for optic neuritis, and how effective are they? | "Optic neuritis, an inflammation of the optic nerve often associated with multiple sclerosis (MS), can be a serious condition. The treatment options and their effectiveness can vary depending on the underlying cause and the individual patient's condition. Here's an overview of common treatments:  1. Corticosteroids: These are often the first line of treatment for optic neuritis. Intravenous (IV) methylprednisolone is commonly used, followed by oral steroids. These can help reduce inflammation and may speed up the recovery of vision. However, steroids do not appear to affect the long-term outcome of vision.  2. Plasma Exchange (Plasmapheresis): In cases where patients do not respond to steroid treatment, plasma exchange might be considered. This treatment involves removing and treating the blood plasma, then returning the blood cells to the body, potentially helping those with severe or steroid-resistant optic neuritis.  3. Immunomodulatory Therapy: For patients with optic neuritis related to MS, disease-modifying therapies may be recommended to decrease the likelihood of future MS-related attacks. This includes various types of medications that modulate or suppress the immune system.  4. Symptomatic Treatment: Pain associated with optic neuritis can be treated with over-the-counter pain relievers. Additionally, strategies to manage symptoms such as vision loss or double vision might involve the use of vision aids or rehabilitation.  The effectiveness of these treatments can vary. While many patients experience a partial or complete recovery of vision, some may have residual deficits. The extent of recovery often depends on the severity of the initial attack and the individual's overall health.  It's important for patients with optic neuritis to work closely with their healthcare provider to determine the best course of action based on their specific circumstances. Regular monitoring and follow-up are crucial to manage the condition effectively." |
| 1. Can optic neuritis be naturally eliminated, and how can I increase the chances of that happening? | "Optic neuritis is an inflammation of the optic nerve, which can cause symptoms such as vision loss, pain with eye movement, and changes in color vision. While the exact cause of optic neuritis is often unknown, it's commonly associated with autoimmune disorders like multiple sclerosis.  In many cases, optic neuritis may improve on its own without treatment. The body's natural healing processes can lead to a partial or even full recovery of vision over several weeks to months. However, the extent of recovery can vary from person to person.  To increase the chances of natural recovery and improve overall eye health, consider the following general recommendations:  1. Maintain a Healthy Lifestyle: Eating a balanced diet rich in antioxidants and essential nutrients can support eye health. Foods high in omega-3 fatty acids, vitamins A, C, and E, and zinc are particularly beneficial.  2. Avoid Smoking: Smoking can negatively affect circulation and overall health, including eye health. Quitting smoking can help in the recovery process.  3. Manage Underlying Conditions: If you have an autoimmune disorder or other health conditions, managing these effectively can contribute to better outcomes in optic neuritis.  4. Rest and Reduce Eye Strain: Giving your eyes adequate rest and avoiding activities that strain them can be helpful during recovery.  5. Regular Monitoring: Regular check-ups with an eye specialist (ophthalmologist) or neurologist are important to monitor the condition and ensure appropriate care.  It's crucial to consult a healthcare professional for a proper diagnosis and personalized advice. They can provide guidance on the best course of action, which may include observation, medical treatment, or other interventions based on your specific case. Optic neuritis can be a serious condition, and professional medical advice is essential for proper management." |
| 1. How can I relieve pain and other symptoms caused by optic neuritis? | "Relieving pain and other symptoms caused by optic neuritis typically involves medical treatment and self-care strategies. Here are some general recommendations:  1. Consult a Healthcare Professional: It's crucial to seek advice from an ophthalmologist, neurologist, or other healthcare professional experienced in treating optic neuritis. They can provide a diagnosis and appropriate treatment plan.  2. Corticosteroids: These medications are often prescribed to reduce inflammation and speed up recovery of vision. They can be administered orally or intravenously.  3. Pain Management: Over-the-counter pain relievers like ibuprofen or acetaminophen may help alleviate pain. For more severe pain, your doctor might prescribe stronger medication.  4. Rest and Recovery: Allowing your body to rest and avoiding strenuous activities can help in recovery.  5. Vision Aids: If you're experiencing vision problems, using aids like magnifying lenses or updated eyeglass prescriptions may help.  6. Healthy Lifestyle: Maintaining a healthy lifestyle, including a balanced diet and regular exercise, can support overall health and potentially aid in recovery.  7. Managing Underlying Conditions: If your optic neuritis is associated with a condition like multiple sclerosis (MS), managing the underlying condition is an important part of treatment.  8. Regular Follow-up: Regular check-ups with your healthcare provider are important to monitor your condition and adjust treatments as necessary.  Remember, these are general guidelines and the specific treatment for optic neuritis can vary depending on the individual case. Always follow the advice and treatment plan provided by your healthcare provider." |
| 1. Is it possible for my vision to fully recover to normal after optic neuritis treatment? | "The prognosis for vision recovery after optic neuritis varies from person to person. Optic neuritis, which is inflammation of the optic nerve, can cause symptoms such as vision loss, pain with eye movement, and changes in color vision. Treatment typically involves corticosteroids to reduce inflammation and speed up the recovery of vision.  Many individuals with optic neuritis experience substantial improvement in their vision over several weeks to months following treatment. However, the extent and speed of recovery can depend on various factors, including the severity of the inflammation, the underlying cause of the optic neuritis, and individual differences in response to treatment.  In some cases, people may recover almost all of their vision, while others may have residual deficits, such as reduced visual acuity, contrast sensitivity, or color vision. A small percentage of individuals might not experience significant recovery.  It's important to have regular follow-ups with a healthcare provider, typically an ophthalmologist or neurologist, to monitor the recovery process and manage any underlying conditions that may be associated with optic neuritis, such as multiple sclerosis or other autoimmune disorders.  Each case is unique, so it's best to discuss your specific situation and prognosis with your healthcare provider. They can provide the most accurate information based on your individual circumstances." |
| 1. What potential risks are associated with corticosteroid therapy for optic neuritis? | "Corticosteroid therapy for optic neuritis can have several potential risks and side effects. While corticosteroids are often used to reduce inflammation and speed up recovery of vision, they are not without risks. Some of the common side effects and risks associated with corticosteroid therapy, especially when used for a prolonged period or in high doses, include:  1. Increased Risk of Infections: Corticosteroids can suppress the immune system, making the body more susceptible to infections.  2. Elevated Blood Sugar Levels: These medications can cause an increase in blood sugar levels, which can be a concern for people with diabetes or those at risk of developing diabetes.  3. Bone Thinning (Osteoporosis): Long-term use of corticosteroids can lead to a decrease in bone density, increasing the risk of fractures.  4. Gastrointestinal Problems: These can include stomach ulcers and bleeding, especially if corticosteroids are taken with certain pain medications like NSAIDs.  5. Mood Swings and Psychological Effects: Some people may experience changes in mood, such as feeling more anxious or depressed.  6. Weight Gain and Fluid Retention: Corticosteroids can cause an increase in appetite and changes in the way the body retains water and salt, leading to weight gain and swelling.  7. Cataracts and Glaucoma: Long-term use of corticosteroids can increase the risk of developing cataracts and glaucoma.  8. Hypertension (High Blood Pressure): These medications can cause an increase in blood pressure.  9. Muscle Weakness: Prolonged use of corticosteroids can lead to muscle weakness.  10. Skin Changes: Thinning of the skin, easy bruising, and slower wound healing are possible.  It's important to note that not everyone will experience these side effects and the benefits of corticosteroid therapy may outweigh the risks in many cases. The decision to use corticosteroids should be made in consultation with a healthcare provider, who can help balance the potential benefits and risks based on the individual's health status and the severity of the optic neuritis." |
| 1. When is the optimal time to begin treating optic neuritis, and what happens if treatment is delayed? | "The optimal time to begin treating optic neuritis is as soon as possible after the diagnosis is confirmed. Early treatment is crucial because optic neuritis can lead to vision loss, and prompt treatment can help minimize the damage and improve the prognosis.  Optic neuritis is an inflammation of the optic nerve, and it can cause symptoms such as vision loss, pain with eye movement, and changes in color vision. The most common treatment for optic neuritis is corticosteroids, which help reduce inflammation and swelling.  If treatment for optic neuritis is delayed, the risk of permanent damage to the optic nerve increases. This can lead to irreversible vision loss or significant impairments in vision. In some cases, even with treatment, some vision loss may be permanent, but early intervention generally improves the likelihood of a better visual outcome.  It's important to note that the underlying cause of optic neuritis also needs to be addressed. Optic neuritis can be associated with conditions like multiple sclerosis or other autoimmune disorders, and managing these underlying conditions is essential for the overall treatment and prognosis.  As always, it's important for individuals to consult with healthcare professionals for personalized advice and treatment options." |

1. **Follow-up and Prevention**

| **Question** | **Response** |
| --- | --- |
| 1. Does optic neuritis commonly recur, and if so, which type has a higher recurrence rate? | "Optic neuritis, an inflammation of the optic nerve that can cause vision loss, does have a risk of recurrence. The likelihood of recurrence can vary based on the underlying cause of the optic neuritis.  There are two main types of optic neuritis:  1. Typical optic neuritis: This is often associated with demyelinating diseases like multiple sclerosis (MS). Recurrence rates can be higher in patients with MS or those who go on to develop MS. In MS, the immune system attacks the protective myelin sheath covering nerve fibers, including the optic nerve, leading to recurrent episodes of inflammation.  2. Atypical optic neuritis: This includes forms of optic neuritis that do not fit the typical presentation or are associated with other systemic conditions like neuromyelitis optica (NMO) or autoimmune disorders. The recurrence rate can vary depending on the specific underlying condition.  Overall, the recurrence rate of optic neuritis is generally higher in patients with underlying autoimmune conditions like MS or NMO. The course of the disease, including the risk of recurrence, can be influenced by various factors such as the type of underlying condition, the initial response to treatment, and the overall management of the systemic disease. Regular monitoring and management by a healthcare professional are crucial in these cases." |
| 1. What preventive measures can I take to avoid developing optic neuritis? | "Preventing optic neuritis involves addressing the risk factors and underlying conditions that can lead to the development of this eye disorder. Optic neuritis is often associated with autoimmune disorders, particularly multiple sclerosis (MS), and other infections or diseases. Here are some general preventive measures:  1. Maintain a Healthy Lifestyle: Adopting a healthy lifestyle can help in preventing diseases that might trigger optic neuritis. This includes eating a balanced diet, exercising regularly, avoiding smoking, and limiting alcohol consumption.  2. Regular Eye Exams: Regular check-ups with an ophthalmologist can help in early detection of any abnormalities in the optic nerve or overall eye health.  3. Manage Chronic Conditions: If you have a chronic condition like diabetes or hypertension, managing these conditions effectively can reduce the risk of complications that might affect your eyes.  4. Monitor and Treat Autoimmune Disorders: If you have an autoimmune disorder like multiple sclerosis, regular monitoring and treatment are crucial. This can help in managing the condition and potentially reduce the risk of optic neuritis.  5. Avoid Infections: Some viral and bacterial infections can lead to optic neuritis. Practicing good hygiene and avoiding exposure to infectious diseases can be helpful.  6. Vaccinations: Stay up to date with vaccinations, as some vaccines can prevent infections that might lead to optic neuritis.  7. Reduce Stress: Chronic stress can have a negative impact on the immune system. Engaging in stress-reducing activities like yoga, meditation, or other relaxation techniques can be beneficial.  8. Consult Healthcare Professionals: If you have concerns or are at a higher risk for optic neuritis, consult with healthcare professionals for personalized advice and monitoring.  Remember, these are general guidelines and may not be applicable to everyone. It's important to consult with healthcare professionals for advice tailored to your specific health needs and circumstances." |
| 1. How should I proceed with follow-up care after experiencing optic neuritis? | "Preventing the recurrence of neuromyelitis optica (NMO), also known as Devic's disease, involves a combination of medical treatments and lifestyle adjustments. Here are some key steps typically recommended by healthcare professionals:  1. Long-term Immunosuppressive Therapy: The mainstay of preventing NMO relapses is the use of medications that suppress or modulate the immune system. Commonly prescribed drugs include rituximab, azathioprine, and mycophenolate mofetil. These medications help reduce the frequency and severity of attacks by suppressing the abnormal immune response that damages the nervous system in NMO.  2. Regular Monitoring: Frequent check-ups with a neurologist are important for monitoring the disease's progression and the effectiveness of treatment. This may include clinical evaluations and MRI scans.  3. Managing Acute Relapses: Prompt treatment of acute relapses with high-dose corticosteroids or plasma exchange (plasmapheresis) can help reduce the severity of attacks.  4. Vaccinations: Some vaccines may be recommended to prevent infections that could potentially trigger a relapse. However, live vaccines should be avoided in people taking immunosuppressive drugs.  5. Lifestyle Changes: Maintaining a healthy lifestyle can support overall well-being. This includes a balanced diet, regular exercise, and avoiding smoking. Managing stress and getting adequate sleep are also important.  6. Treatment of Associated Conditions: Addressing other health conditions such as pain, bladder and bowel problems, and depression, which are common in NMO patients, can improve quality of life and potentially reduce the risk of relapses.  7. Avoidance of Trigger Factors: If specific triggers for relapses are identified (like certain infections), steps should be taken to avoid these triggers.  It's important to note that treatment plans can vary significantly from one person to another, and they should be personalized based on individual health needs and the specifics of the disease in each case. Always consult with a healthcare professional for the best course of action." |
| 1. What steps can be taken to prevent the recurrence of neuromyelitis optica? | "After experiencing optic neuritis, follow-up care is crucial to monitor your recovery and manage any underlying conditions. Here are some general guidelines for follow-up care:  1. Regular Check-ups with an Ophthalmologist or Neurologist: Schedule regular appointments with your eye doctor (ophthalmologist) or a neurologist. They will monitor your vision and the health of your optic nerve.  2. Visual Field Testing: Your doctor may recommend regular visual field tests to assess any changes in your peripheral vision.  3. Optical Coherence Tomography (OCT): OCT scans can be used to measure the thickness of the retinal nerve fiber layer, which can be affected by optic neuritis.  4. MRI Scans: If you haven't already had one, your doctor may recommend an MRI scan of your brain to check for lesions that might indicate multiple sclerosis (MS) or other neurological conditions.  5. Managing Underlying Conditions: If an underlying condition like MS or an autoimmune disorder is associated with your optic neuritis, it's important to follow your doctor's treatment plan for that condition.  6. Medications: Your doctor may prescribe medications to manage symptoms or treat an underlying condition.  7. Visual Rehabilitation: If you have persistent vision problems, visual rehabilitation with a specialist might be helpful.  8. Lifestyle Changes: Maintaining a healthy lifestyle, including regular exercise, a balanced diet, and avoiding smoking, can be beneficial.  9. Be Aware of Symptoms: Stay alert to any changes in your vision or other symptoms and report them to your doctor promptly.  10. Emotional Support: Dealing with vision loss can be challenging. Consider seeking support from counseling or support groups.  Remember, these are general guidelines, and your specific follow-up care should be tailored to your individual needs and medical history. Always follow the advice and instructions of your healthcare provider." |
| 1. In the event of a recurrence of neuromyelitis optica, what actions should I take? | "In the event of a recurrence of neuromyelitis optica (NMO), it's crucial to take immediate and appropriate actions, as NMO can lead to serious complications. Here are the general steps you should follow:  1. Seek Medical Attention Immediately: If you suspect a recurrence of NMO, contact your healthcare provider or go to the emergency room as soon as possible. NMO attacks can lead to severe symptoms and complications, so prompt medical intervention is essential.  2. Follow Your Treatment Plan: If you have a pre-established treatment plan for NMO, follow it closely. This might include taking certain medications or following specific protocols during an attack.  3. Medication Administration: Treatment of NMO relapses often involves high doses of corticosteroids, such as intravenous methylprednisolone, to reduce inflammation. Plasmapheresis (plasma exchange) or intravenous immunoglobulin (IVIG) may also be used in certain cases, especially if there's no response to steroid treatment.  4. Monitor Symptoms: Keep track of your symptoms and report any changes to your healthcare provider. This includes new or worsening symptoms, such as visual changes, muscle weakness, or changes in sensation.  5. Rest and Self-Care: During a relapse, it's important to rest and take care of yourself. Avoid overexertion and ensure you get adequate rest to support your body's recovery.  6. Follow-up Appointments: Keep all follow-up appointments with your healthcare providers. These appointments are important for monitoring your condition and adjusting treatment as necessary.  7. Lifestyle and Dietary Considerations: Follow any dietary or lifestyle recommendations provided by your healthcare provider, which may help in managing symptoms and overall health.  8. Support Network: Lean on your support network, including family, friends, and support groups for individuals with NMO. Emotional and practical support can be invaluable during a relapse.  Remember, every person's experience with NMO is unique, and the specific actions you should take may vary based on your individual health situation. Always consult with your healthcare provider for personalized advice and treatment." |
| 1. What potential consequences might arise from multiple recurrences of neuromyelitis optica? | "Neuromyelitis optica (NMO), also known as Devic's disease, is a rare neurological disorder that primarily affects the eyes and spinal cord. Multiple recurrences of NMO can lead to several potential consequences, some of which can be severe. These include:  1. Visual Impairment or Blindness: NMO can cause inflammation of the optic nerve (optic neuritis), leading to visual problems. Repeated episodes can result in partial or complete loss of vision.  2. Motor Function Impairment: Recurrent attacks can damage the spinal cord, leading to weakness or paralysis of the limbs. This can range from mild weakness to severe paralysis.  3. Sensory Disturbances: Patients may experience numbness, tingling, or pain due to spinal cord involvement.  4. Bladder and Bowel Dysfunction: Spinal cord lesions can interfere with bladder and bowel control, leading to incontinence or constipation.  5. Respiratory Problems: Severe attacks, particularly those affecting the cervical spine, can impair respiratory muscles, potentially leading to life-threatening respiratory failure.  6. Fatigue and Pain: Chronic pain and fatigue are common in NMO patients, affecting quality of life.  7. Cognitive and Emotional Effects: While less common than in multiple sclerosis, cognitive impairment and emotional disturbances like depression can occur in NMO.  8. Increased Disability: Each attack can lead to an accumulation of disability, which may become irreversible over time.  It's important to note that NMO is a heterogeneous condition, and the severity and types of symptoms can vary greatly among individuals. Early diagnosis and treatment are crucial in managing NMO and preventing or minimizing these potential consequences. Treatments typically involve immunosuppressive therapies to reduce the frequency and severity of attacks. Regular monitoring and supportive care are also important aspects of managing the condition." |
| 1. Can individuals with neuromyelitis optica safely receive vaccinations? | "Individuals with neuromyelitis optica (NMO), a rare autoimmune disorder affecting the central nervous system, can generally receive vaccinations, but this should be decided on a case-by-case basis. The concern with vaccinations in autoimmune conditions like NMO is the potential risk of triggering or exacerbating the autoimmune response. However, the benefits of vaccination often outweigh these risks, especially considering the severe consequences of preventable infectious diseases.  Live vaccines are usually avoided in individuals with autoimmune disorders who are on immunosuppressive therapy, as they could pose a higher risk. Non-live vaccines are generally considered safer and are often recommended. These include vaccines for influenza, pneumococcal disease, hepatitis, and others.  It's important for individuals with NMO to consult with their healthcare provider to discuss the specific risks and benefits of vaccinations in their particular case, considering their current health status, treatment regimen, and the type of vaccine in question. The healthcare provider can offer personalized advice and make recommendations based on the latest medical guidelines and research." |
