# Supplementary material for: Evaluation and comparison of large language models’ responses to questions related optic neuritis
Source: Front Med (Lausanne). 2025 Jun 25;12:1516442. doi: 10.3389/fmed.2025.1516442 (PMC12238082; doi:10.3389/fmed.2025.1516442)
Supplement: Supplementary file 5 [file Table_5.docx]

**Table S5: Readability Scales and Explication**

| **Scale** | **Formula** | **Explication** |
| --- | --- | --- |
| Flesh-Kincaid gradel level | (0.39 x [total words/total sentences]) + (11.8 x [total syllables/total words]) - 15.59 | This scale calculates the U.S. school grade level needed to understand the text by analyzing the average sentence length and syllable count per word. |
| Gunning Fog index | (0.4 x [total words/total sentences]) + (100 x [total complex words/total words]) | It measures the number of years of formal education required to understand a text on the first reading, based on sentence length and the proportion of complex words. |
| SMOG index | 1.0430 (sqrt [complex words] x [30/number of sentences]) + 3.1291 | This index estimates the years of education necessary to comprehend a text, using the count of polysyllabic words and total sentences to gauge complexity. |
| Coleman-Liau index | 5.89 x [total characters/total words]) - (0.3 x [total sentences/total words]) - 15.8 | It predicts the grade level required to understand a text based on the number of characters per word and words per sentence, focusing on letter count rather than syllables. |

Abbreviation: SMOG, Simple Measure of Gobbledygoo
