# Supplementary material for: Evaluation and comparison of large language models’ responses to questions related optic neuritis
Source: Front Med (Lausanne). 2025 Jun 25;12:1516442. doi: 10.3389/fmed.2025.1516442 (PMC12238082; doi:10.3389/fmed.2025.1516442)
Supplement: Supplementary file 6 [file Table_6.docx]

**Table S6: Readability Score of Content from LLMs and Website**

| **Readability scale** | **Score, mean(SD)** | | | | |
| --- | --- | --- | --- | --- | --- |
|  | **Claude-2** | **ChatGPT-3.5** | **Google Bard** | **ChatGPT-4.0** | **Website** |
| Flesch-Kincaid grade Level | 11.56 (1.82) | 13.75 (1.57) | 12.61 (2.07) | 13.87 (1.77) | 9.78 (2.29) |
| Gunning Fog index | 14.56 (2.42) | 17.67 (1.96) | 15.70 (2.13) | 17.68 (2.33) | 12.68 (2.88) |
| SMOG index | 10.47 (1.63) | 12.62 (1.31) | 10.92 (1.47) | 12.76 (1.65) | 8.82 (1.72) |
| Coleman-Liau index | 13.92 (1.94) | 15.16 (1.67) | 15.60 (2.69) | 14.69 (1.54) | 12.09 (2.69) |

Abbreviation: SMOG, Simple Measure of Gobbledygoo
