# Supplementary material for: Evaluation and comparison of large language models’ responses to questions related optic neuritis
Source: Front Med (Lausanne). 2025 Jun 25;12:1516442. doi: 10.3389/fmed.2025.1516442 (PMC12238082; doi:10.3389/fmed.2025.1516442)
Supplement: Supplementary file 7 [file Table_7.docx]

**Table S7: Row Score and Rating of each LLM-Chatbots**

| **Category** | **Question**  **No.** | **Claude-2** | | | **ChatGPT-3.5** | | | **Google Bard** | | | **ChatGPT-4.0** | | |
| --- | --- | --- | --- | --- | --- | --- | --- | --- | --- | --- | --- | --- | --- |
|  |  | **Average**  **Total Score (Acc)**  **Max: 9** | **Final**  **Rating** | **Average**  **Score (Comp)**  **Max: 3** | **Average**  **Total Score (Acc)**  **Max: 9** | **Final**  **Rating** | **Average**  **Score (Comp)**  **Max: 3** | **Average**  **Total Score (Acc)**  **Max: 9** | **Final**  **Rating** | **Average**  **Score (Comp)**  **Max: 3** | **Average**  **Total Score (Acc)**  **Max: 9** | **Final**  **Rating** | **Average**  **Score (Comp)**  **Max: 3** |
| **General** | 1 | 4.67 | Marginal | - | 6.67 | Marginal | - | 8.33 | Excellent | 3.00 | 6.33 | Marginal | - |
|  | 2 | 5.67 | Excellent | 2.33 | 8.00 | Excellent | 2.33 | 7.00 | Marginal | - | 8.00 | Excellent | 2.67 |
|  | 3 | 8.00 | Excellent | 3.00 | 7.33 | Marginal | - | 4.67 | Deficient | - | 7.33 | Marginal | - |
|  | 4 | 5.33 | Marginal | - | 6.33 | Excellent | 2.33 | 6.00 | Marginal | - | 7.67 | Excellent | 2.67 |
|  | 5 | 6.67 | Marginal | - | 7.67 | Excellent | 2.33 | 8.00 | Excellent | 2.67 | 8.00 | Excellent | 2.33 |
|  | 6 | 7.33 | Marginal | - | 7.33 | Marginal | - | 9.00 | Excellent | 2.00 | 9.00 | Excellent | 2.67 |
| **Diagnose** | 1 | 7.00 | Marginal | - | 8.33 | Excellent | 3.00 | 7.67 | Excellent | 2.33 | 8.00 | Excellent | 2.67 |
|  | 2 | 6.67 | Marginal | - | 7.33 | Marginal | - | 7.67 | Excellent | 2.33 | 7.33 | Excellent | 3.00 |
|  | 3 | 8.00 | Excellent | 2.67 | 8.67 | Excellent | 3.00 | 8.00 | Excellent | 3.00 | 7.33 | Marginal | - |
|  | 4 | 7.00 | Excellent | 2.33 | 7.33 | Marginal | - | 6.67 | Marginal | - | 7.67 | Excellent | 2.67 |
|  | 5 | 6.67 | Marginal | - | 7.00 | Marginal | - | 8.67 | Excellent | 3.00 | 7.00 | Marginal | - |
| **Treatmemt** | 1 | 6.67 | Marginal | - | 7.67 | Excellent | 2.33 | 7.67 | Excellent | 3.00 | 7.00 | Marginal | - |
|  | 2 | 7.33 | Excellent | 3.00 | 6.67 | Excellent | 2.67 | 5.00 | Deficient | - | 6.33 | Excellent | 2.67 |
|  | 3 | 4.33 | Deficient | - | 7.67 | Excellent | 2.33 | 8.00 | Excellent | 2.67 | 8.33 | Excellent | 3.00 |
|  | 4 | 4.33 | Deficient | - | 7.33 | Marginal | - | 8.33 | Excellent | 3.00 | 7.33 | Marginal | - |
|  | 5 | 6.33 | Excellent | 2.33 | 7.33 | Marginal | - | 8.67 | Excellent | 2.33 | 9.00 | Excellent | 2.67 |
|  | 6 | 6.67 | Excellent | 2.67 | 7.33 | Marginal | - | 5.00 | Marginal | - | 9.00 | Excellent | 2.67 |
| **Follow-up**  **and Prevention** | 1 | 7.33 | Marginal | - | 5.33 | Excellent | 1.67 | 7.67 | Excellent | 3.00 | 5.67 | Marginal | - |
|  | 2 | 6.33 | Marginal | - | 6.33 | Marginal | - | 8.33 | Excellent | 3.00 | 7.00 | Marginal | - |
|  | 3 | 7.00 | Marginal | - | 7.33 | Marginal | - | 7.33 | Marginal | - | 8.33 | Excellent | 2.67 |
|  | 4 | 7.00 | Marginal | - | 6.33 | Excellent | 2.33 | 8.33 | Excellent | 2.67 | 8.00 | Excellent | 2.67 |
|  | 5 | 6.67 | Marginal | - | 7.33 | Marginal | - | 7.00 | Marginal | - | 8.33 | Excellent | 3.00 |
|  | 6 | 7.00 | Marginal | - | 7.00 | Marginal | - | 8.00 | Marginal | - | 7.00 | Marginal | - |
|  | 7 | 4.67 | Marginal | - | 7.33 | Marginal | - | 7.00 | Marginal | - | 8.00 | Excellent | 3.00 |
