# Supplementary material for: Evaluation and comparison of large language models’ responses to questions related optic neuritis
Source: Front Med (Lausanne). 2025 Jun 25;12:1516442. doi: 10.3389/fmed.2025.1516442 (PMC12238082; doi:10.3389/fmed.2025.1516442)
Supplement: Supplementary file 8 [file Table_8.docx]

**Table S8: Comprehensiveness Scores for Responses Rated as “Excellent”**

| **LLM** | **Response comprehensiveness** | |
| --- | --- | --- |
|  | **n** | **Median** |
| **Claude-2** | 7 | 2.67 |
| **ChatGPT-3.5** | 10 | 2.33 |
| **Google Bard** | 14 | 2.84 |
| **ChatGPT-4.0** | 15 | 2.67 |
