# Supplementary material for: Evaluation and comparison of large language models’ responses to questions related optic neuritis
Source: Front Med (Lausanne). 2025 Jun 25;12:1516442. doi: 10.3389/fmed.2025.1516442 (PMC12238082; doi:10.3389/fmed.2025.1516442)
Supplement: Supplementary file 9 [file Table_9.docx]

**Table S9: Main Findings**

| **Category** | **Main Finding** |
| --- | --- |
| **Readability** | Responses generated by all LLM-Chatbots require at least a college-level reading ability. |
| **Accuracy** | ChatGPT-4.0 received the highest accuracy score, followed by Google Bard, and then ChatGPT-3.5 and Claude-2. Notably, 8.3% of responses from both Claude-2 and Google Bard were rated as "Deficient." |
| **Comprehensiveness** | The comprehensiveness scores for "Excellent" responses generated by the four LLM-Chatbots were comparable, with no significant differences observed. |
